# Carbohydrate deacetylase, a key enzyme in oxidative chitin degradation, is evolutionarily linked to amino acid deacetylase

**DOI:** 10.1016/j.jbc.2025.108420

**Published:** 2025-03-18

**Authors:** Jing-Ping Wang, Xiang-Ming Zhao, Xiao-Lei Liu, Wen-Xin Jiang, Chao Gao, Hai-Yan Cao, Hai-Tao Ding, Qi-Long Qin, Xiu-Lan Chen, Yu-Zhong Zhang, Ping-Yi Li

**Affiliations:** 1 State Key Laboratory of Microbial Technology, Shandong University, Qingdao, China; 2 MOE Key Laboratory of Evolution and Marine Biodiversity, Frontiers Science Center for Deep Ocean Multispheres and Earth System & College of Marine Life Sciences, Ocean University of China, Qingdao, China; 3 Laboratory for Marine Biology and Biotechnology, Qingdao Marine Science and Technology Center & Laoshan Laboratory, Qingdao, China; 4 Antarctic Great Wall Ecology National Observation and Research Station, Polar Research Institute of China, Ministry of Natural Resources, Shanghai, China; 5 Marine Biotechnology Research Center, State Key Laboratory of Microbial Technology, Shandong University, Qingdao, China

**Keywords:** carbohydrate deacetylase, catalysis, evolution, oxidative chitin degradation, structural adaption

## Abstract

The microbial oxidative cleavage of chitin, the second most abundant biopolymer in nature, generates a substantial amount of oxidized amino sugar, 2-(acetylamino)-2-deoxy-D-gluconic acid (GlcNAc1A). The catabolism of GlcNAc1A is key to the oxidative chitin degradation pathway. However, the molecular mechanism and evolution underlying this pathway remain elusive. Here, we target OngB, which initiates the GlcNAc1A catabolism, to explore the molecular mechanism driving the evolution of this process. We characterized *Pp*OngB (the OngB from *Pseudoalteromonas prydzensis* ACAM 620) and its homologs as specific deacetylases for GlcNAc1A and solved the structures of WT *Pp*OngB and its inactive mutant in complex with GlcNAc1A. Structural, mutational, and biochemical analyses revealed that *Pp*OngB utilizes a D-aminoacylase-like (β/α)_8_-barrel fold to deacetylate GlcNAc1A in a metal-dependent manner. *Pp*OngB and its homologs significantly differ from other known carbohydrate de-*N*-acetylases in sequences, substrate specificities, and structures. Phylogenetic analysis indicated that *Pp*OngB and its homologs represent a new carbohydrate de-*N*-acetylase family, forming a sister group of D-aminoacylases involved in the catabolism of *N*-acetyl-D-amino acids. Further structural analysis suggested that GlcNAc1A deacetylases likely evolved from an ancestral D-aminoacylase, undergoing structural and electrostatic modifications in the catalytic cavity to hydrolyze GlcNAc1A. This study provides insights into the catalytic mechanism and the divergent evolution of GlcNAc1A deacetylases, advancing our understanding of oxidative chitin degradation.

Chitin is the second most abundant biopolymer after cellulose in nature, providing a significant carbon and nitrogen source for bacterial growth (1, 2, 3). It is an insoluble linear polysaccharide of β-1,4 linked *N*-acetyl-D-glucosamine (GlcNAc). The crystalline structure and insolubility of chitin, often in conjunction with its embedding in complex matrices, contribute to its resistance to microbial hydrolysis. Since 2010, oxidative degradation initiated by lytic polysaccharide monooxygenases (LPMOs) has been recognized as essential to the efficient bioconversion of chitin and other recalcitrant polysaccharides (4, 5, 6, 7, 8). LPMOs create “nicks” on the surfaces of crystalline polysaccharides, promoting further degradation by traditional hydrolytic enzymes (4, 5, 6, 7, 8).

LPMOs require O_2_ or H_2_O_2_ as the final electron acceptor during catalysis (4, 8, 9). Molecular oxygen was introduced in the Earth’s atmosphere during the Great Oxidation Event, approximately 2.4 to 2.1 billion years ago (10, 11, 12). The presence of LPMOs likely emerged after molecular oxygen became available in Earth’s early environment (13). To date, all known chitin-active LPMOs only oxidize chitin to produce C1-oxidized chitooligosaccharides, which feature a terminal 2-(acetylamino)-2-deoxy-D-gluconic acid (GlcNAc1A) (6, 14, 15, 16, 17). GlcNAc1A is a hallmark intermediate of the oxidative chitin utilization pathway, differing from GlcNAc, a product from chitin degradation through the hydrolytic pathway, by both chemical structure and charge (Fig. 1). Recently, we reported the GlcNAc1A catabolic pathway in the Antarctic marine chitinolytic bacterium *Pseudoalteromonas prydzensis* ACAM 620 based on transcriptomic, gene KO and enzymatic product analyses (18). In this pathway, GlcNAc1A undergoes sequential conversions into 2-keto-3-deoxy phosphogluconate (KDG-6P) by enzymes OngB, OngC, and KdgK (Fig. 1). This pathway, however, diverges from the typical phosphorylation-initiated catabolism of GlcNAc and other monosaccharides (19, 20, 21) by directly deacetylating and deaminating GlcNAc1A without phosphorylation, resembling the *N*-acetyl-D-amino acid catabolism. The catabolism of GlcNAc1A may have evolved later, following the emergence of LPMOs. Despite its important role in oxidative chitin degradation, our understanding of the molecular mechanism and evolution of the GlcNAc1A catabolism remains quite limited.

**Figure 1:**
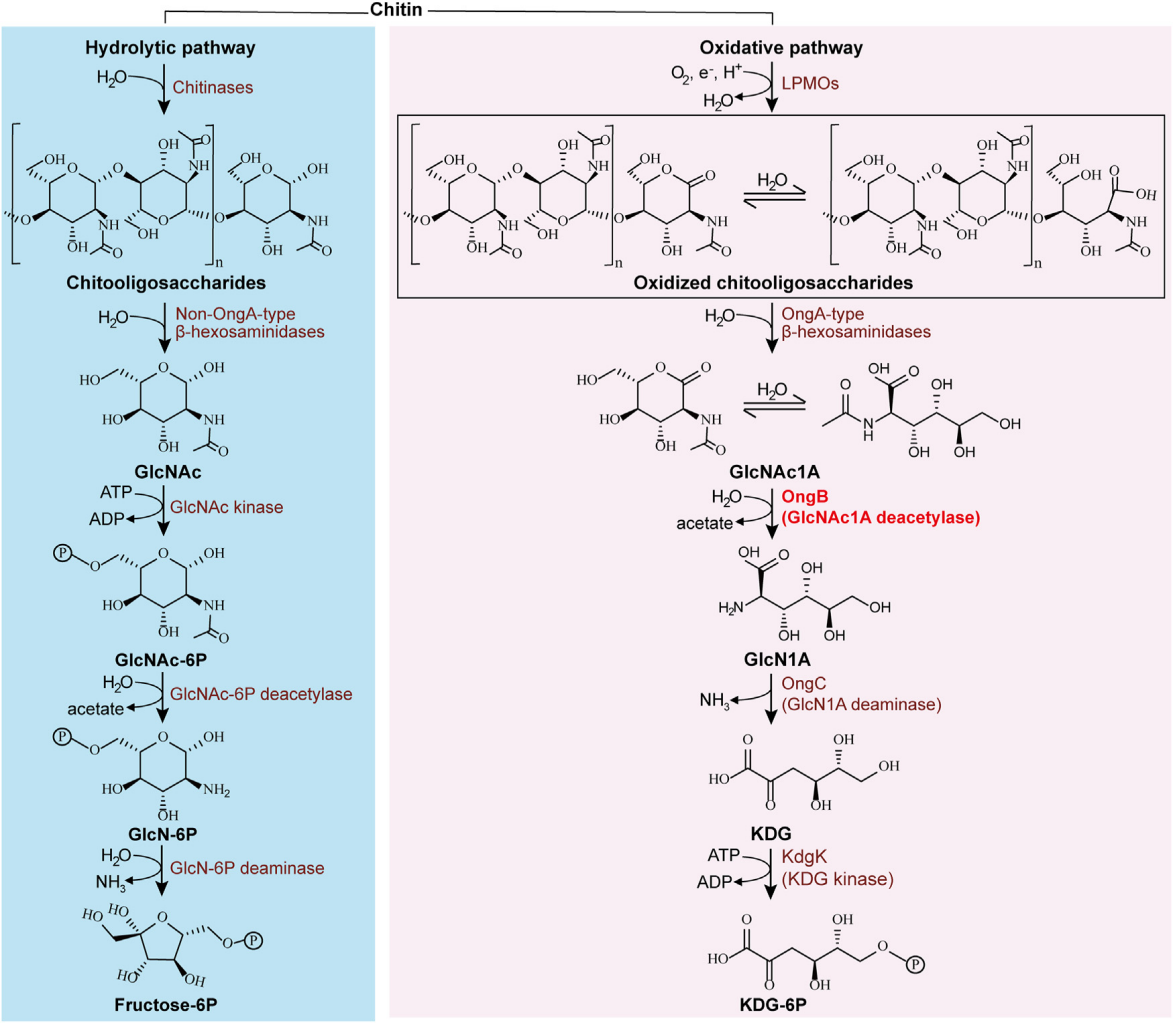
**Chitin degradation pathways.** The hydrolytic chitin utilization pathway begins with chitinases, which hydrolyze polymeric chitin into chitooligosaccharides. The catabolism of GlcNAc starts with phosphorylation, followed by deacetylation and deamination, ultimately producing fructose-6P. The oxidative chitin utilization pathway is initiated by LPMOs, which oxidize polymeric chitin into chitooligosaccharides with a terminal oxidized sugar, GlcNAc1A. The resulting oxidized chitooligosaccharides (1,5-δ-lactone) exist in pH-dependent equilibrium with their hydrates (aldonic acid). GlcNAc1A is then deacetylated and deaminated directly to produce KDG without phosphorylation activation, resembling the catabolism of *N*-acetyl-D-amino acids rather than other monosaccharides. OngB, the GlcNAc1A deacetylase that initiates the GlcNAc1A catabolism, is colored in *red*. GlcN, D-glucosamine; KDG, 2-keto-3-deoxygluconate; GlcNAc, *N*-acetyl-D-glucosamine; GlcNAc1A, 2-(acetylamino)-2-deoxy-D-gluconic acid; LPMO, lytic polysaccharide monooxygenase.

OngB, functioning as a carbohydrate de-*N*-acetylase, catalyzes the first step of the GlcNAc1A catabolic pathway by deacetylating GlcNAc1A to form 2-(amino)-2-deoxy-D-gluconic acid (GlcN1A) (Fig. 1). The deacetylation of GlcNAc1A is a key step due to its initiation of the GlcNAc1A pathway. Therefore, further studies on OngB are needed to gain insights into the molecular mechanism and evolution of the GlcNAc1A catabolism.

In the CAZy database, carbohydrate de-*N*-acetylases are grouped into carbohydrate esterase (CE) families 4, 9, 11, 14, and 18 according to sequence similarities and structural folds (22). CE4 enzymes, characterized by a (β/α)_7_-barrel fold, deacetylate structural polysaccharides including chitin, poly-β-1,6-GlcNAc, and peptidoglycan (23, 24, 25). CE9 enzymes, with a (β/α)_8_-barrel structure, catalyze the deacetylation of *N*-acetyl-D-glucosamine-6-phosphate (GlcNAc-6P) in the GlcNAc catabolic pathway (26, 27). CE11 enzymes, which have a two-layer sandwich fold, are involved in lipid A biosynthesis by deacetylating UDP-3-*O*-acyl-GlcNAc (28, 29). CE14 enzymes, with an α/β fold, deacetylate the GlcNAc moiety of oligosaccharides (30), while the only characterized CE18 enzyme is a *N*-acetyl-galactosamine deacetylase with a (β/α)_7_-barrel fold distantly related to CE4 enzymes (31). These carbohydrate de-*N*-acetylases are all metal-dependent hydrolases, sharing a common metal-assisted acid/base mechanism (32, 33, 34). OngB, however, uniquely functions as a GlcNAc1A deacetylase in the GlcNAc1A catabolic pathway and shares no sequence homology with other known carbohydrate de-*N*-acetylases, suggesting that its structure and catalytic mechanism may differ from those previously reported. Due to the lack of structural information, the catalytic mechanism of GlcNAc1A deacetylases remains unknown. We found that OngB shares significant sequence homology to bacterial D-aminoacylases involved in the *N*-acetyl-D-amino acid catabolism. It is considered that both L- and D-amino acids existed on primal earth before the emergence of life (35, 36, 37, 38, 39). Thus, it is reasonable to hypothesize that OngB may have evolved from D-aminoacylases, and it is intriguing to uncover the underlying mechanism for driving a D-aminoacylase-like amidohydrolase, OngB, to evolve to hydrolyze the amino sugar, GlcNAc1A.

In this study, we characterized the OngB from *P. prydzensis* ACAM 620 (*Pp*OngB) and solved the crystal structures of WT *Pp*OngB and its inactive mutant in complex with GlcNAc1A. Based on structural and mutational analyses, the catalytic mechanism of *Pp*OngB was illustrated. Phylogenetic and structural analyses suggested that *Pp*OngB and its homologs define a new family of carbohydrate de-*N*-acetylases, adopting a D-aminoacylase-like (β/α)_8_-barrel fold. Furthermore, we explained the evolutionary divergence that enables *Pp*OngB to function as a GlcNAc1A deacetylase. This study provides important clues for understanding the evolution of oxidative chitin degradation, particularly regarding the GlcNAc1A catabolism.

## Results and discussion

### Biochemical characterization of *Pp*OngB

Among characterized enzymes, *Pp*OngB is most closely related to the metal-dependent D-aminoacylases from *Alcaligenes faecalis* (*Af*Dam) (40) and *Bordetella bronchiseptica* (*Bb*Dam) (41), with sequence identities of 46% and 42%, respectively. D-aminoacylases belong to the amidohydrolase superfamily, a structure-based cluster of enzymes that contain a sturdy and versatile triosephosphate isomerase (TIM)-like (β/α)_8_-barrel fold encompassing the catalytic active site (42, 43, 44). Based on metal-binding ligands, amidohydrolases are classified into nine subtypes (42, 43, 44). Both *Af*Dam and *Bb*Dam are subtype V amidohydrolases, characterized by a Cys/His/His metal-binding site and a catalytic Asp residue (40, 41, 44). Sequence alignment indicated that *Pp*OngB contains a typical metal-binding site of subtype V amidohydrolases, formed by Cys97, His222, and His252, with Asp368 likely serving as the catalytic residue (Fig. S1), suggesting that *Pp*OngB is a member of subtype V amidohydrolases.

**Figure 2:**
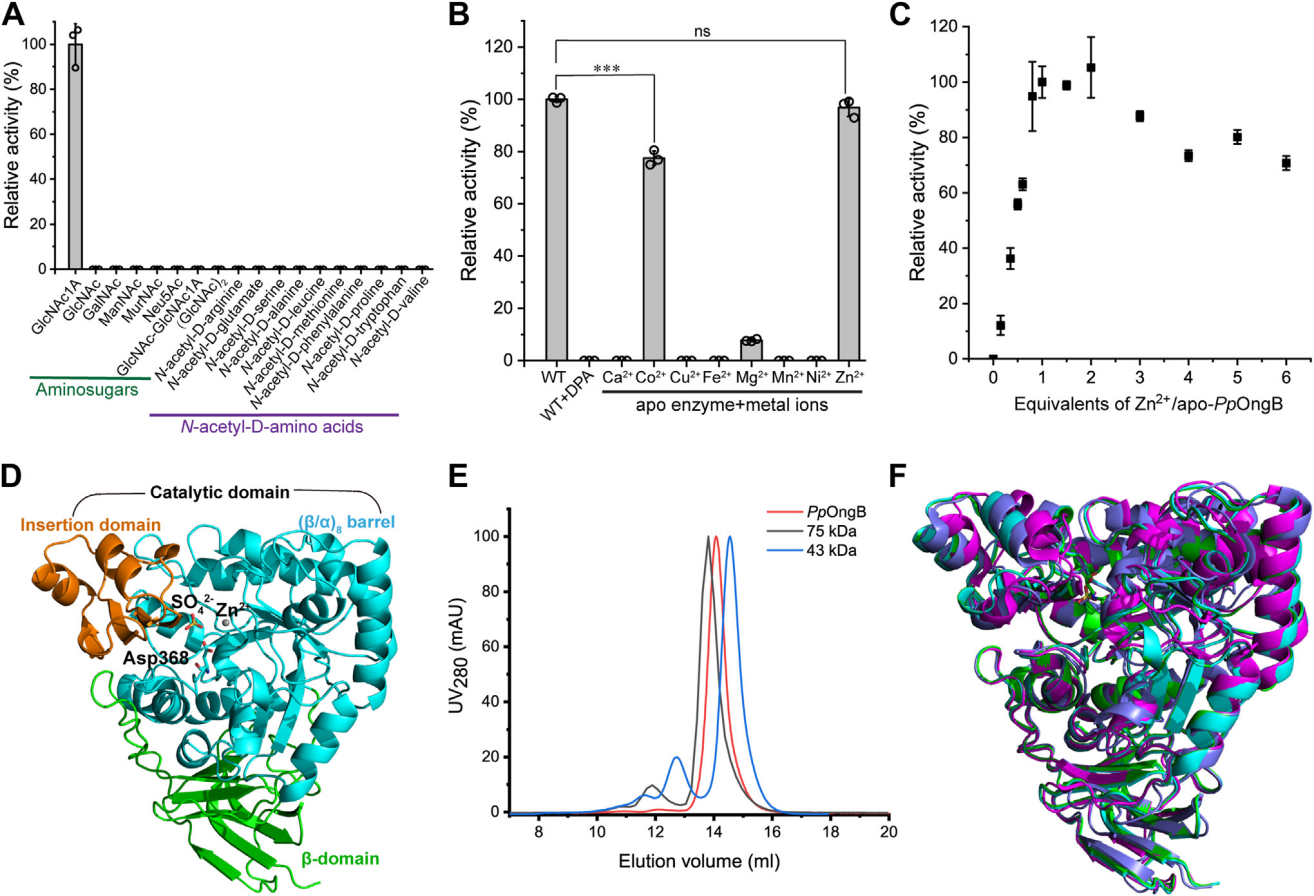
**Biochemical characterization and overall structural analysis of *Pp*OngB.***A*, substrate specificity analysis of *Pp*OngB at 30 °C in 10 mM Bis-Tris buffer (pH 7.5) using 10 mM substrate and 0.75 μM enzyme. The specific activity of *Pp*OngB against GlcNAc1A (7.4 U/mg) was taken as 100%. The data shown in the graph are from triplicate experiments (mean ± S.D.). *B*, relative activities of the apo *Pp*OngB with different metal ions. WT *Pp*OngB was treated with 10 mM DPA to obtain the apo enzyme. The apo *Pp*OngB (1.5 μM) was incubated with 5 μM metal ions for 0.5 h at 4 °C, and then the activity was determined against 10 mM GlcNAc1A in 10 mM metal-free Bis-Tris buffer (pH 7.5) at 30 °C. The enzyme activity of the WT *Pp*OngB was set to 100%. The data shown in the graph are from triplicate experiments (mean ± S.D.) and were analyzed using an independent two-sample *t* test. ns indicates no statistically significant difference with a *p* value of 0.193, and ∗∗∗ indicates *p* < 0.001. *C*, reconstitution of the apo *Pp*OngB with varying ratios of ZnCl_2_. The apo *Pp*OngB (1.5 μM) was incubated with varying ratios of ZnCl_2_ for 20 h at 4 °C and then the activity was determined against 10 mM GlcNAc1A in 10 mM metal-free Bis-Tris buffer (pH 7.5) at 30 °C. The activity of the apo *Pp*OngB with a 1:1 molar ratio of zinc was normalized to 100%. The data shown in the graph are from triplicate experiments (mean ± S.D.). *D*, structure of *Pp*OngB in one asymmetric unit. The catalytic domain of *Pp*OngB binds one Zn ion and one sulfate molecule. The catalytic residue Asp368 is shown as *sticks*. *E*, gel filtration analysis of recombinant *Pp*OngB and markers. *Pp*OngB monomer has a calculated molecular mass of 53.0 kDa. The two protein size markers are ovalbumin (43 kDa) and conalbumin (75 kDa). *F*, superimposition of the WT *Pp*OngB, the *Pp*OngB-GlcNAc1A complex and D-aminoacylases. *Pp*OngB (*cyan*), the *Pp*OngB-GlcNAc1A complex (*green*), *Af*Dam (*magenta*) and *Bb*Dam (*slate*) are shown in different colors. GalNAc, *N*-acetyl-D-galactosamine; ManNAc, *N*-acetyl-D-mannosamine; MurNAc, *N*-acetylmuramic acid; Neu5Ac, *N*-acetylneuraminic acid; DPA, dipicolinate; GlcNAc, *N*-acetyl-D-glucosamine; GlcNAc1A, 2-(acetylamino)-2-deoxy-D-gluconic acid.

To investigate the substrate preference of *Pp*OngB, we compared the activities of *Pp*OngB, *Af*Dam, and *Bb*Dam against a range of amino sugars and *N*-acetyl-D-amino acids (Figs. 2 and S2). Consistent with previous reports (40, 41), both *Af*Dam and *Bb*Dam displayed deacetylase activities for *N*-acetyl-D-amino acids but were inactive toward GlcNAc1A or other amino sugars (Fig. S2, *C* and *D*), demonstrating their high substrate specificity for *N*-acetyl-D-amino acids. In contrast, *Pp*OngB exhibited hydrolytic activity exclusively toward GlcNAc1A, with no detectable activity for any *N*-acetyl-D-amino acids (Fig. 2*A*), underscoring its strong substrate specificity for GlcNAc1A. We also examined two *Pp*OngB homologs, *Pa*OngB from *Pseudoalteromonas arabiensis* and *Pf*OngB from *Pseudoalteromonas flavipulchra* DSM 14401, which share 76% and 57% sequence identities with *Pp*OngB, respectively. Similar to *Pp*OngB, both *Pa*OngB and *Pf*OngB deacetylated only GlcNAc1A (Fig. S2, *A* and *B*), further indicating that *Pp*OngB, together with its homologs, functions as a carbohydrate deacetylase rather than a D-aminoacylase.

*Pp*OngB exhibited the highest activity at 40 °C and remained stable at temperatures below 30 °C (Fig. S3, *A* and *B*). *Pp*OngB was highly active at pH 7.0 to 7.5 (Fig. S3*C*). *Pp*OngB appeared to be a metalloenzyme because 10 mM dipicolinate (DPA) completely abolished its activity against GlcNAc1A (Fig. 2*B*).

### Overall structure of *Pp*OngB

To elucidate the catalytic mechanism of GlcNAc1A deacetylases, we solved the crystal structure of WT *Pp*OngB at a resolution of 1.77 Å resolution. Using molecular replacement with an AlphaFold2-generated model of *Pp*OngB as the initial template, we refined the structure, with relevant statistics summarized in Table S1. Electron density was absent for the N-terminal residues Met1-Ser6 and C-terminal residues Leu483-Asp489. Crystals of *Pp*OngB belongs to the P3_1_21 space group, with one molecule per asymmetric unit (Fig. 2*D*). Gel filtration analysis confirmed that the WT *Pp*OngB is monomeric in solution (Fig. 2*E*).

The overall structure of *Pp*OngB is similar to those of subtype V amidohydrolases, most closely resembling the structures of D-aminoacylases *Af*Dam (Protein Data Bank (PDB) code 1M7J) (40, 45) and *Bb*Dam (PDB code 3GIP) (41), with RMSDs of 0.81 Å (413 Cα atoms) and 1.16 Å (425 Cα atoms), respectively (Fig. 2*F*). Like these D-aminoacylases (40, 41), *Pp*OngB comprises a catalytic domain (Gly63-Tyr415) and a smaller β-domain (Gln7-Pro62 and Arg416-Ser482) (Fig. 2*D*). The β-domain consists of eight β-sheets from both the N and C termini. The catalytic domain adopts a (β/α)_8_-barrel fold, with an insertion domain (Ser290-His347) containing four β-sheets and two α-helices inserted after β7. Reported carbohydrate de-*N*-acetylases and D-aminoacylases are all metal-dependent enzymes (32, 33, 34, 40, 41, 45). Although zinc is present in most proteins, other metals like iron, cadmium, cobalt, and copper can occasionally occupy the active site (32, 33, 34, 40, 41, 45). Electron density mapping of *Pp*OngB indicates a metal ion within the active site. Inductively coupled plasma-mass spectrometry (ICP-MS) revealed zinc as the predominant metal, occupying ∼78% of the *Pp*OngB molecules, while iron and nickel each accounted for ∼5%. Furthermore, treatment of *Pp*OngB with 10 mM DPA resulted in a complete loss in activity, which could be almost fully restored by the addition of Zn^2+^ (Fig. 2*B*). These results indicated that *Pp*OngB is a metalloenzyme with a zinc ion bound in the active site. A conserved aspartate, critical for catalysis in *Af*Dam, *Bb*Dam, and other subtype V amidohydrolases (40, 41, 44), corresponds to Asp368 in *Pp*OngB. Asp368, located on the loop immediately following β8 from the (β/α)_8_-barrel and 5.15 Å away from the bound zinc in the active site, is most likely the key catalytic residue of *Pp*OngB (Fig. 2*D*). In addition, the active site of the WT *Pp*OngB also binds a sulfate molecule (Fig. 2*D*).

To investigate the interaction of *Pp*OngB with the GlcNAc1A substrate, we mutated Asp368 to alanine. This resulted in full loss of enzymatic activity and enabled cocrystallization of *Pp*OngB with GlcNAc1A. The crystals grew under the same conditions used for the WT protein. We then determined the crystal structure of *Pp*OngB in complex with GlcNAc1A (the *Pp*OngB-GlcNAc1A complex) at a resolution of 2.29 Å (Table S1). This complex formed monomers in the crystal and in solution, with overall structure closely matching that of the WT form (a RMSD of 0.24 Å across 465 Cα atoms) (Fig. 2*F*).

### *Pp*OngB is a mononuclear amidohydrolase

**Figure 3:**
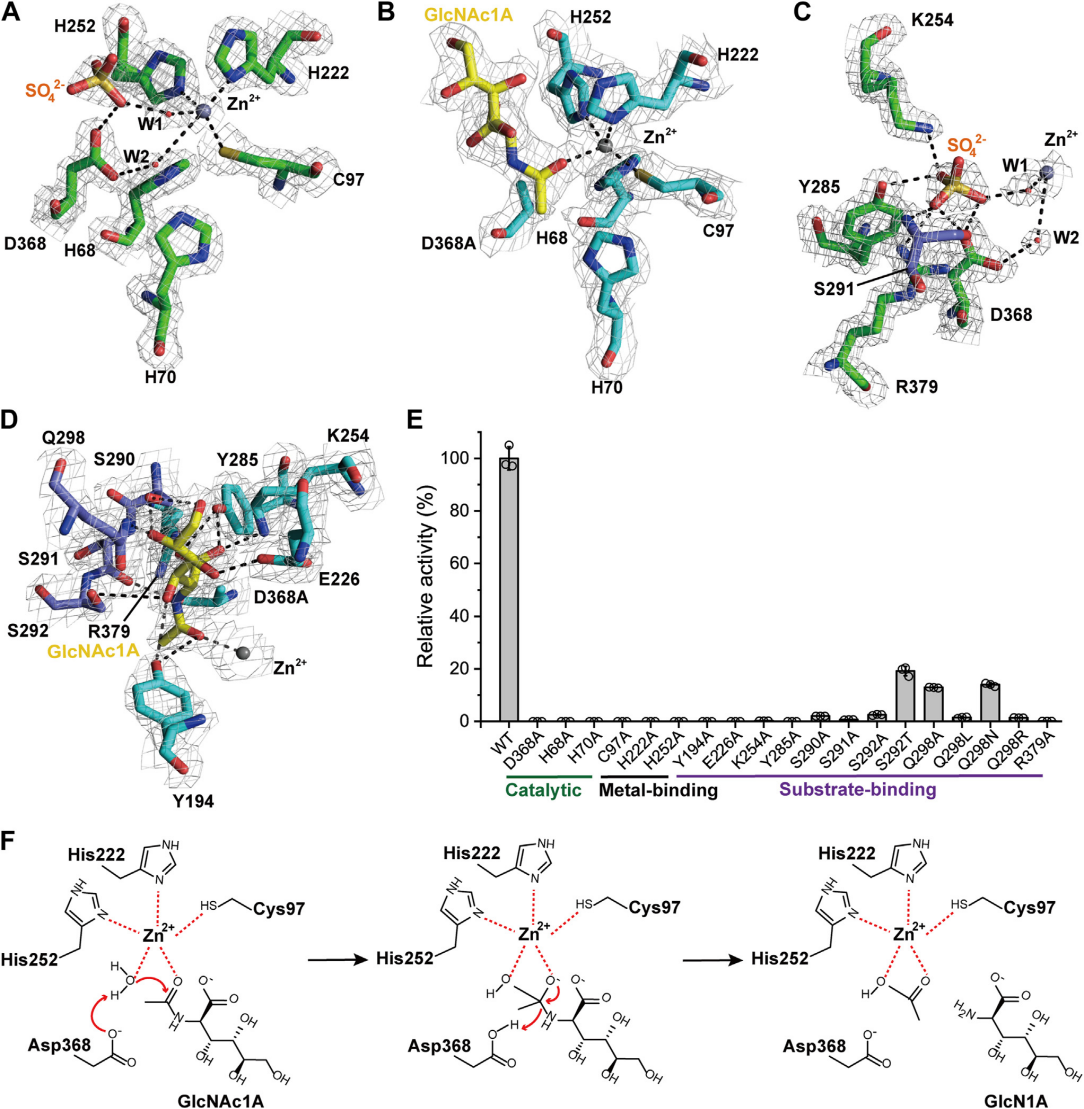
***Pp*OngB deacetylates GlcNAc1A in a metal-dependent manner.***A*, active site of the WT *Pp*OngB with one Zn ion and one sulfate molecule bound. Sulfate is colored in *orange*. *B*, active site of the *Pp*OngB-GlcNAc1A complex with one Zn ion and one GlcNAc1A molecule bound. GlcNAc1A is colored in *yellow*. *C*, detailed structure of the WT *Pp*OngB. Sulfate is colored in *orange*. Residues involved in sulfate binding are shown as *sticks*, with residues from the (β/α)_8_-barrel colored in *green* and those from the insertion domain in *slate*. *D*, detailed structure of the *Pp*OngB-GlcNAc1A complex. GlcNAc1A is colored in *yellow*. Residues involved in GlcNAc1A binding are shown as *sticks*, with residues from the (β/α)_8_-barrel colored in *cyan* and those from the insertion domain in *slate*. In (*A*)–(*D*), the 2*F*_*o*_*-F*_*c*_ densities for key residues, meatal ions, water molecules, sulfate, and GlcNAc1A are contoured in *gray* at 1.0 σ, and possible hydrogen bonds are represented by *dashed lines*. *E*, enzymatic activities of *Pp*OngB and its mutants. The activities were determined at 30 °C in 10 mM Bis-Tris buffer (pH 7.5) with 50 mM GlcNAc1A as the substrate. The specific activity of WT *Pp*OngB (12.4 U/mg) was taken as 100%. The graph shows data from triplicate experiments (mean ± S.D.). *F*, proposed catalytic mechanism for *Pp*OngB hydrolyzing GlcNAc1A into acetate and GlcN1A. GlcNAc1A, 2-(acetylamino)-2-deoxy-D-gluconic acid.

In the structure of WT *Pp*OngB, a single zinc ion is exclusively coordinated at the β site within the active site (Fig. 3). The zinc ion is coordinated by Cys97, His222, and His252, as well as a sulfate molecule mediated by a water molecule (W1), along with a weakly interacting water molecule (W2) (3.46 Å) that is hydrogen bonded to the catalytic Asp368 (Fig. 3*A*). A five-coordinate zinc ion has also been observed in other zinc-dependent hydrolases (30, 46). In subtype V amidohydrolases, a Zn-coordinated water is essential for hydrolysis and is also hydrogen bonded to the conserved catalytic aspartate residue (42, 44). The distance between water molecule W1 and the catalytic Asp368 exceeds 4.25 Å, suggesting that no hydrogen bond is formed between them. Furthermore, the hydrolysis of GlcNAc1A by *Pp*OngB is not affected by sodium sulfate at concentrations up to 600 mM (Fig. S4), indicating that water molecule W1 is not the hydrolytic water molecule. Instead, water molecule W2 not only coordinates the zinc ion but also forms a hydrogen bond with the side chain of the catalytic Asp368 (Fig. 3*A*), suggesting that this water molecule functions as the hydrolytic water molecule.

In the *Pp*OngB-GlcNAc1A complex, the zinc ion remains bound at the β site, directly coordinated not only by Cys97, His222, and His252 but also by the bound GlcNAc1A molecule (Fig. 3*B*). Water molecule W2 is absent in the complex (Fig. 3*B*), likely due to the mutation of the hydrophilic Asp368 to the smaller hydrophobic alanine residue, rather than to the binding of the substrate GlcNAc1A. Mutating any of these residues to alanine led to an extremely low or no enzymatic activity (Fig. 3*E*), underscoring their essential roles in zinc ion binding. An additional potential binding site, the α site, is also present in *Pp*OngB, which is surrounded by residues His68, His70, Cys79, and Asp368 (Fig. 3*A*). Previous studies have shown that, under high concentrations of metal ions such as zinc, copper, and cadmium, the α site in *Af*Dam can bind a second metal ion, leading to attenuated enzyme activity (45). However, no electron density corresponding to a bound metal ion at this α site was observed in either the WT or complexed structures of *Pp*OngB, as our crystallization experiments were conducted without the addition of external metal ions. To determine whether the active site in *Pp*OngB requires one or two metal ions to be fully active, the apo enzyme was reconstituted by adding varying ratios of zinc ions. The maximum catalytic activity was observed at approximately a 1:1 molar ratio of zinc (Fig. 2*C*), demonstrating that *Pp*OngB requires only a single zinc ion for its catalytic activity. Notably, the spatial proximities of His68-Nδ1 (3.35 Å) and His70-Nε2 (3.78 Å) to the catalytic Asp368-Oδ1 suggest that these residues may facilitate the catalysis of *Pp*OngB. Mutation of either His68 or His70 in *Pp*OngB to alanine abolished enzymatic activity completely (Fig. 3*E*), supporting their roles in catalysis. Based on the binding site(s) of the catalytically essential metal ion(s), amidohydrolases are classified into four types: αβ-binuclear, α-mononuclear, β-mononuclear, and metal-independent subsets (47). Our results indicate that *Pp*OngB falls within the β-mononuclear subset. Furthermore, *Pp*OngB possesses a subtype V metal-binding site composed of Cys97, His222, and His252, which are conserved among *Af*Dam, *Bb*Dam, and other subtype V amidohydrolases (Fig. S5).

### The substrate-binding mode of *Pp*OngB

In the WT *Pp*OngB, a sulfate molecule from the crystallization solution is bound in the active site (Fig. 3*A*). This sulfate, coordinated with the zinc ion *via* a water molecule, may occupy the binding position of the substrate carboxylate group. Sulfate is hydrogen bonded to the side chains of Lys254, Tyr285, Asp368, and Arg379 from the (β/α)_8_-barrel as well as to the side and main chains of Ser291 from the insertion domain (Fig. 3*C*), suggesting the roles of these residues in substrate binding.

In the aqueous environment, the amino sugar GlcNAc1A exists in equilibrium between two forms: the fused-ring 1,5-δ-lactone and the open-chain aldonic acid. Under alkaline conditions, GlcNAc1A in its fused-ring form spontaneously converts to the open-chain aldonic acid (4, 14). In buffers with pH values ranging from 6.0 to 8.0, the *K*_m_ values of *Pp*OngB were similar (Fig. S6*A*), suggesting that *Pp*OngB has comparable affinities for both forms of GlcNAc1A. However, the *k*_cat_ values of *Pp*OngB were influenced by pH, with the maximum at pH 7.0 (Fig. S6*B*). In the structure of the *Pp*OngB–GlcNAc1A complex, a clear electron density for GlcNAc1A in the open-chain aldonic acid form is visible in the catalytic cavity of *Pp*OngB (Fig. 3*B*). The amide nitrogen atom of GlcNAc1A is spatially close to the catalytic D368A residue, while its amide oxygen atom coordinates the zinc ion (Fig. 3*B*). GlcNAc1A is further stabilized by hydrogen bond interactions (Fig. 3*D*). Its amide oxygen forms a hydrogen bond with the side chain of Tyr194, and the amide nitrogen forms a hydrogen bond with the main-chain CO group of Ser291. The α-carboxylate group aligns with the position of the sulfate molecule, forming hydrogen bonds with side chains of Lys254, Tyr285, and Arg379 and the main-chain CO and NH groups of Ser291. The C3-hydroxy group is hydrogen bonded to Ser292, while the C4-hydroxy group interacts with Glu226. The C5- and C6-hydroxy groups form hydrogen bonds with the side chain of Ser290, and the C5-hydroxy group is further stabilized by hydrogen bonding with the amide nitrogen of Gln298. Among these residues, Ser290, Ser291, Ser292, and Gln298 belong to the insertion domain, while the others are from the (β/α)_8_-barrel, mainly in its protruding loops (Fig. 3*D*). Additional hydrophobic interactions, involving Leu116 and Leu370, stabilize GlcNAc1A by interacting with the methyl group of its acetamido moiety. Structural superimposition suggested that no significant conformational change was observed for the metal-binding and substrate-binding residues of the WT *Pp*OngB and its complex (Fig. S5). Alanine substitution of the identified residues led to complete or significant loss of enzymatic activity (Fig. 3*E*), highlighting their crucial roles in substrate binding. Specifically, Q298A and Q298N mutations severely decreased the *k*_cat_ values of *Pp*OngB and its affinity to GlcNAc1A (Table 1), further confirming the role of Gln298 in substrate binding *via* its side chain. CD spectroscopy analysis showed that the secondary structures of the mutants exhibit little deviation from that of WT *Pp*OngB (Fig. S7), indicating that the changes in enzymatic activity and kinetic parameters of the mutants result from residue substitution rather than structural changes.

**Table 1:** Kinetic parameters of *Pp*OngB, *Pa*OngB, *Pf*OngB, and *Pp*OngB mutants^a^

Type	Enzyme	*V*_max_ (μmol/min/mg)	*K*_m_ (mM)	*k*_cat_ (s^−1^)	*k*_cat_/*K*_m_ (mM^−1^ s^−1^)^b^
Wild type	*Pp*OngB	17.4 ± 1.5	9.8 ± 0.8	15.4 ± 1.3	1.6
	*Pa*OngB	6.3 ± 0.3	9.1 ± 0.8	5.5 ± 0.3	0.6
	*Pf*OngB	13.4 ± 0.8	17.2 ± 1.0	11.9 ± 0.7	0.7
*Pp*OngB mutant	Q298A	7.7 ± 0.7	59.7 ± 5.0	6.8 ± 0.6	0.11 (6.9%)
	Q298N	9.4 ± 0.3	53.4 ± 2.5	8.3 ± 0.3	0.16 (10.0%)
	S292T	1.3 ± 0.1	54.2 ± 1.8	1.1 ± 0.1	0.02 (1.3%)

a Reactions were conducted in triplicate in 10 mM Bis-Tris buffer (pH 7.5) at 30 °C using GlcNAc1A as the substrate over a concentration range of 2 to 120 mM.

b Percentages in parentheses were calculated relative to wild-type *Pp*OngB.

### The catalytic mechanism of *Pp*OngB

Based on our structural and biochemical results on *Pp*OngB, along with known catalytic mechanisms of other carbohydrate de-*N*-acetylases (26, 34) and D-aminoacylases (40, 41), we propose the mechanism for *Pp*OngB to catalyze the deacetylation of GlcNAc1A (Fig. 3*F*). *Pp*OngB contains a subtype V mononuclear metal-binding site composed of Cys97, His222, and His252 to coordinate a water molecule in the active site. Upon substrate binding, GlcNAc1A enters the catalytic cavity and is stabilized mainly through hydrogen bond interactions with nine hydrophilic/polar residues from both the (β/α)_8_-barrel and the insertion domain. These interactions orient the amide bond of GlcNAc1A close to the zinc ion and the coordinated water molecule. Catalysis begins with Asp368 acting as a base, abstracting a proton from the water molecule to generate a nucleophilic hydroxide. This hydroxide ion attacks the amide carbon atom of GlcNAc1A, forming a tetrahedral oxyanion intermediate. Asp368 then switches its role to function as a catalytic acid, donating a proton to the amide nitrogen of the intermediate, which facilitates the cleavage of the amide bond and results in the release of acetate and GlcN1A as products (Fig. 3*F*). In addition, residues His68 and His70, conserved in *Pp*OngB homologs and other subtype V amidohydrolases (Figs. 3*A* and S1), likely enhance the proton transfer capacity of Asp368 during the catalytic cycle.

### *Pp*OngB and its homologs represent a new carbohydrate de-*N*-acetylase family with a D-aminoacylase-like fold

**Figure 4:**
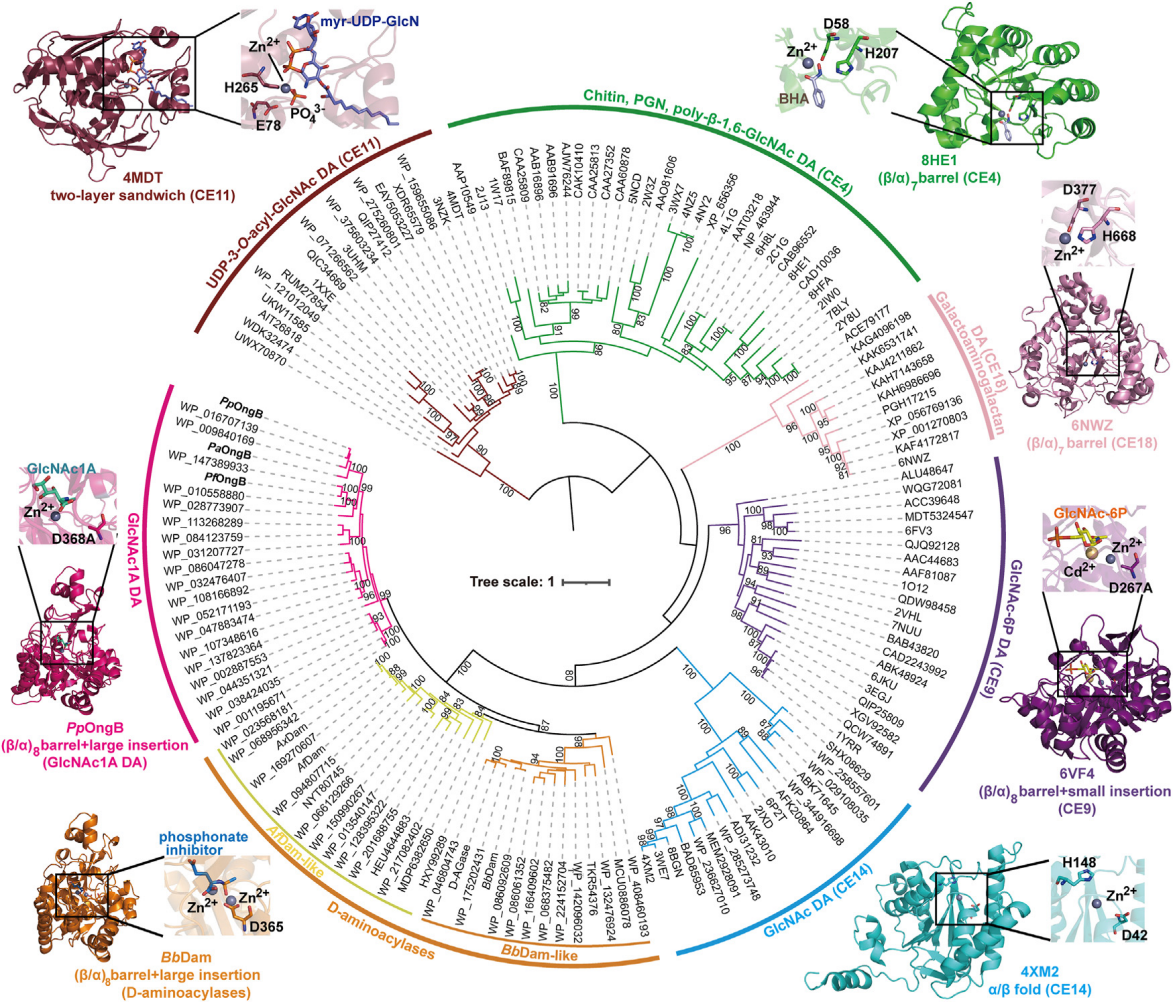
**Phylogenetic and structural analyses of *Pp*OngB and other carbohydrate de-*N*-acetylases.** The tree, including 150 protein sequences, was constructed by the maximum-likelihood method using IQ-TREE. Bootstrap analysis of 1000 replicates is conducted and values above 80 are shown. A more detailed tree with source strain shown for each sequence is provided in Fig. S8. A representative structural fold is shown for the catalytic domains of each carbohydrate de-*N*-acetylase family. Close-ups of the active sites show catalytic residues, metal ions, and bound substrates/products/inhibitors. DA, de-*N*-acetylase; BHA, benzohydroxamic acid; myr-UDP-GlcN, UDP-(3-*O*-(R-3-hydroxymyristoyl))-glucosamine; PGN, peptidoglycan.

To date, all characterized carbohydrate de-*N*-acetylases are metal-dependent hydrolases (32, 33, 34), which are distributed in five families including CE4, CE9, CE11, CE14, and CE18 (22). These enzymes adopt different structural folds and display different substrate specificities (Fig. 4). Among these families, CE4, CE9, and CE14 enzymes are involved in chitin degradation. CE4 enzymes with a (β/α)_7_-barrel fold deacetylate structural polysaccharides such as chitin, poly-β-1,6-GlcNAc, and peptidoglycan (23, 24, 25). CE14 enzymes adopt an α/β fold to deacetylate the GlcNAc moiety of oligosaccharides (30), whereas CE9 enzymes, featuring a (β/α)_8_-barrel structure, specially deacetylate GlcNAc-6P (26, 27, 47). Phylogenetic analysis using either IQ-TREE or RAxML revealed that *Pp*OngB and its homologs form a distinct group separate from known carbohydrate de-*N*-acetylases, showing a closer relationship to D-aminoacylases than to other carbohydrate de-*N*-acetylases (Figs. 4, S8 and S9). Moreover, *Pp*OngB and its homologs exhibit unique substrate specificity, selectively deacetylating GlcNAc1A, a hallmark intermediate of the oxidative chitin utilization pathway (Figs. 2, 4 and S2).

**Figure 5:**
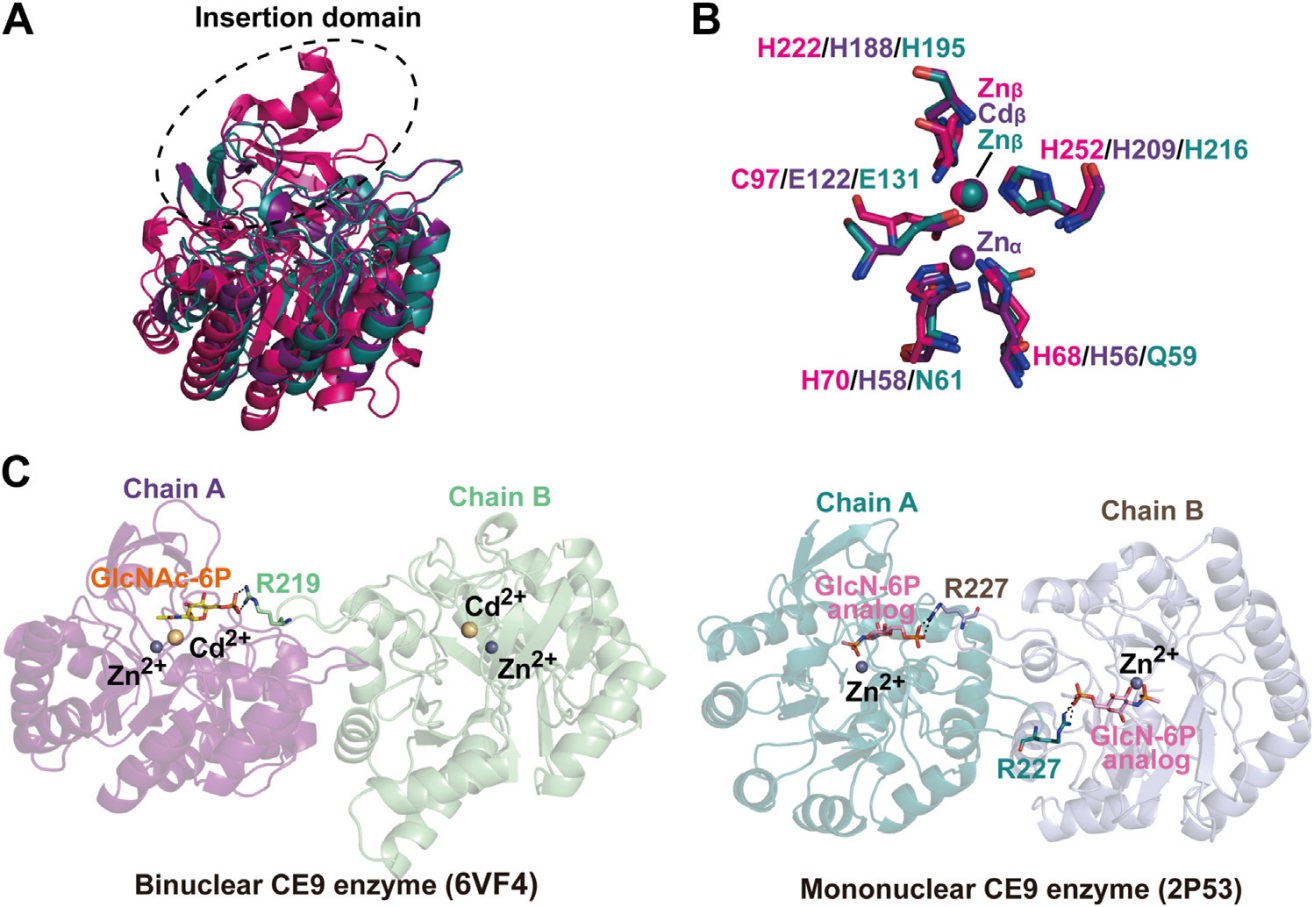
**Comparative structural analyses of *Pp*OngB and CE9 family GlcNAc-6P deacetylases.***A*, superimposition of the catalytic domains of *Pp*OngB (*magenta*) and CE9 family GlcNAc-6P deacetylases from *Mycobacterium tuberculosis* (*purple*) and *E. coli* (*teal*). Insertion domains of *Pp*OngB and CE9 enzymes are marked by a *black oval*. *B*, superimposition of the metal-binding residues in *Pp*OngB (*magenta*) and CE9 family GlcNAc-6P deacetylases from *Mycobacterium tuberculosis* (*purple*) and *E. coli* (*teal*). *C*, dimerization of GlcNAc-6P deacetylases from the CE9 family. In CE9 enzymes, the bound substrate/product analog in monomer A is stabilized by an arginine residue from monomer B. GlcN-6P, D-glucosamine-6-phosphate; CE, carbohydrate esterase; GlcNAc-6P, *N*-acetyl-D-glucosamine-6-phosphate.

Among carbohydrate de-*N*-acetylases, only CE9 enzymes adopt the (β/α)_8_-barrel fold (26, 27, 34), but *Pp*OngB has no sequence homology with CE9 enzymes. While both share the central (β/α)-fold, significant structural differences exist between *Pp*OngB and CE9 enzymes with RMSDs exceeding 10.9 Å. *Pp*OngB possesses a large insertion domain composed of four β-sheets and two α-helices, whereas CE9 enzymes contain small ones comprising only two β-sheets (Fig. 5*A*). Both *Pp*OngB and CE9 enzymes are metal-dependent amidohydrolases, but their metal-binding sites differ. *Pp*OngB and its homologs have a subtype V mononuclear metal-binding site composed of Cys/His/His, whereas the CE9 family include both mononuclear and binuclear members with a subtype IV metal-binding site comprising Glu/His/His (plus a HxH motif) (Fig. 5*B*). In *Pp*OngB, the insertion domain is essential for substrate binding in its monomeric form (Fig. 3). In contrast, all characterized CE9 enzymes function as dimers (26, 27, 48), requiring a conserved Arg residue from one monomer to stabilize the phosphate group of the GlcNAc-6P substrate from the other monomer without the involvement of the insertion domain (Fig. 5*C*).

Together, *Pp*OngB and its homologs significantly differ from other carbohydrate de-*N*-acetylases in sequences, substrate specificities, and structures. Therefore, we suggest that *Pp*OngB and its homologs represent a new CE family with a D-aminoacylase-like fold.

### Structure comparison of *Pp*OngB and D-aminoacylases to reveal the structural basis for the formation of the carbohydrate deacetylase activity of *Pp*OngB

**Figure 6:**
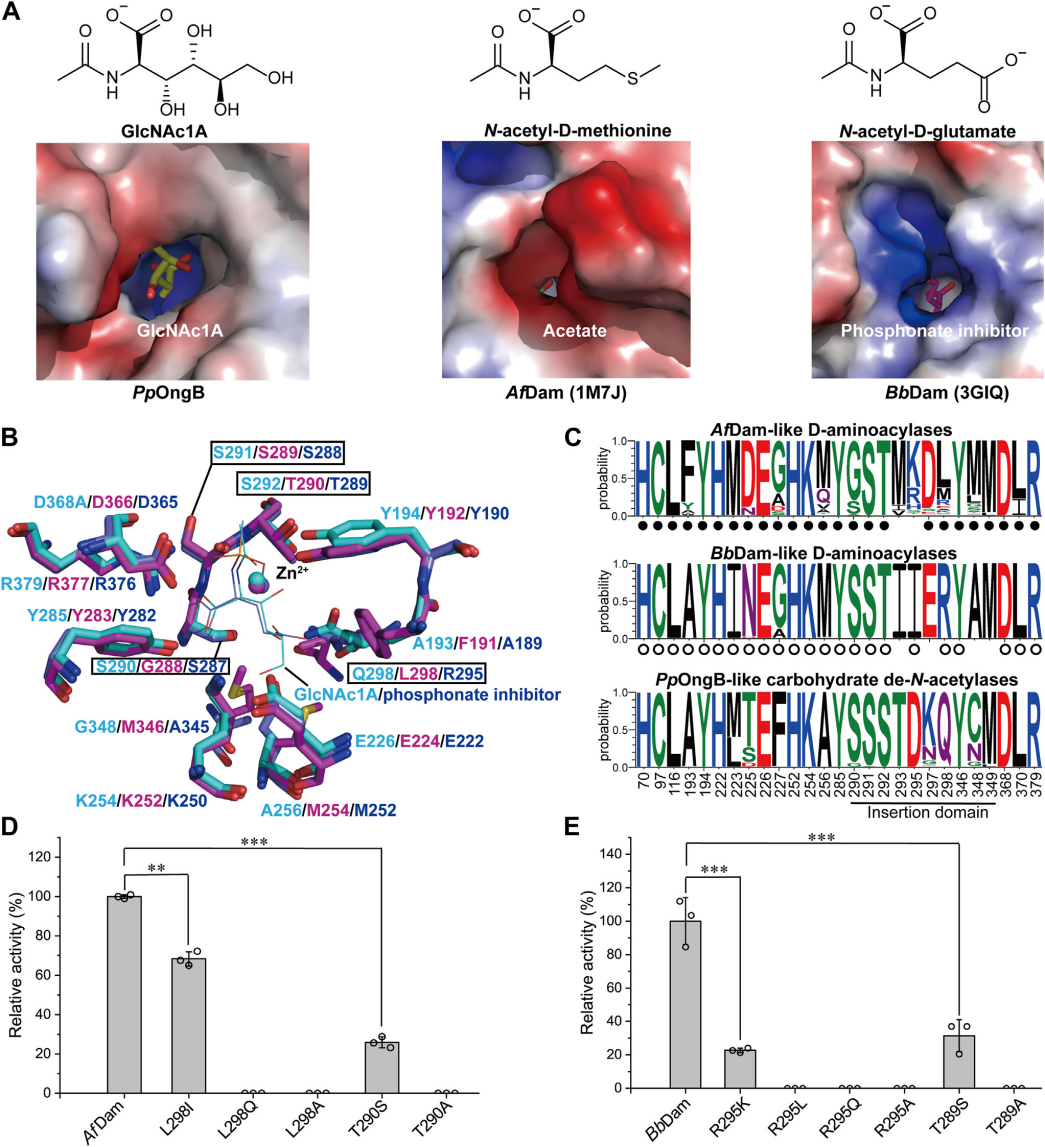
**Comparative structural analyses of *Pp*OngB and D-aminoacylases.***A*, electrostatic surfaces of the catalytic cavities of *Pp*OngB and D-aminoacylases, with positively charged regions shown in *blue* and negatively charged regions in *red*. The bound substrate/product/inhibitor is shown as *sticks* in different colors. The structural formula of the optimal substrate catalyzed by *Pp*OngB, *Af*Dam, and *Bb*Dam is also shown at the *top*. *B*, superposition of the active sites of *Pp*OngB (*cyan*), *Af*Dam (*magenta*), and *Bb*Dam (*slate*). Residues from the insertion domain are boxed. The GlcNAc1A in *Pp*OngB is shown as *cyan lines*, and the phosphate inhibitor (*N*-phosphonomethyl-D-glutamic acid) from *Bb*Dam in *slate lines*. For clarity, only the metal ions at the β site are shown. *C*, an overview of the conservation of residues forming the catalytic cavities of *Pp*OngB and its homologs and D-aminoacylases. Residues are numbered according to *Pp*OngB. Residues forming the catalytic cavities of *Af*Dam-like and *Bb*Dam-like D-aminoacylases are marked by *solid* and *open circles*, respectively. *D*, enzymatic activities of *Af*Dam and its mutants. The activities were determined at 40 °C in 10 mM Bis-Tris buffer (pH 7.0) with 10 mM *N*-acetyl-D-methionine as the substrate. *E*, enzymatic activities of *Bb*Dam and its mutants. The activities were determined at 30 °C in 50 mM Hepes buffer (pH 8.0) with 10 mM *N*-acetyl-D-glutamate as the substrate. In (*D*) and (*E*), the graphs show data from triplicate experiments (mean ± S.D.), which were analyzed using an independent two-sample *t* test. ∗∗, *p* < 0.01; ∗∗∗, *p* < 0.001. GlcNAc1A, 2-(acetylamino)-2-deoxy-D-gluconic acid.

Phylogenetic analysis revealed that *Pp*OngB and its homologs form a separate group from D-aminoacylases (Figs. 4, S8 and S9), suggesting the divergent evolution of GlcNAc1A deacetylases from D-aminoacylase ancestors. Although *Pp*OngB and D-aminoacylases share a similar topological structure, they differ in the shape and electrostatic profile of their catalytic cavities (Fig. 6). For these enzymes, the zinc ion and the catalytic residue Asp are all located near the bottom of their catalytic cavities. *Af*Dam has a hydrophobic catalytic cavity with a negatively charged narrow opening, suitable for accommodating *N*-acetyl derivatives of hydrophobic amino acids, especially D-methionine (Fig. 6*A*). *Bb*Dam has a positively charged catalytic cavity near the opening, suited for stabilizing the negatively charged γ-carboxylate group of *N*-acetyl-D-glutamate (Fig. 6*A*). *Pp*OngB, however, presents a positively charged catalytic cavity with a wide opening, accommodating GlcNAc1A and its four hydroxyl groups at the C3 to C6 positions (Fig. 6*A*). Compared to the narrow catalytic cavity mouth in *Af*Dam (bound to two acetate molecules), the cavities in *Pp*OngB and *Bb*Dam adopt a more open conformation, with loop structures that affect accessibility. In *Pp*OngB, these loops correspond to residues Leu223-Ala229 from the (β/α)_8_-barrel (loop 1) and Leu294-Asp304 from the insertion domain (loop 2) (Fig. S10). The loop 2 of *Af*Dam is closer to its loop 1, resulting in a more closed cavity, while *Pp*OngB and *Bb*Dam maintain an open conformation regardless of substrate or inhibitor binding.

In *Pp*OngB, residues Leu116, Tyr194, Tyr285, Lys254, Ser291, Asp368, Leu370, and Arg379 are involved in the coordination of the acetamido and α-carboxylate groups of GlcNAc1A (Fig. 3). Their counterparts in *Af*Dam are involved in the binding of two acetate molecules, which occupy the positions of the acetate product and the α-carboxylate group of the substrate, respectively (40). These residues as well as metal ligands are strictly conserved in *Pp*OngB-like, *Af*Dam-like, and *Bb*Dam-like sequences (Fig. 6, *B* and *C*), suggesting a shared mechanism between GlcNAc1A deacetylases and D-aminoacylases for anchoring substrate acetamido and α-carboxylate groups. However, differences arise in substrate specificity due to variation in residues that bind other parts of the substrate. *Af*Dam features a hydrophobic cavity, with residues Phe191, Met254, Leu298, Met346, and nearby hydrophobic/aromatic residues, suited for hydrophobic side chains of *N*-acetyl derivatives of D-methionine and other hydrophobic D-amino acids (40). Among these residues, except for Leu298, the others are largely conserved in *Bb*Dam. In *Bb*Dam, the counterpart of hydrophobic Leu298 in *Af*Dam is replaced by a hydrophilic Arg295 to coordinate the γ-carboxylate group of *N*-acetyl-D-glutamate (41). By contrast, *Pp*OngB is occupied by small/polar residues corresponding to Ala193, Ala256, Gln298, and Gly348 at the equivalent positions of the catalytic cavity (Fig. 6*B*), creating a slightly hydrophilic environment favorable for the hydroxyl groups of GlcNAc1A. Gln298, conserved in *Pp*OngB and its homologs, stabilizes the C5-hydroxy group of GlcNAc1A *via* hydrogen bonding, which is replaced by a conserved hydrophobic Leu in *Af*Dam-like sequences and a conserved basic Arg in *Bb*Dam-like sequences (Fig. 6, *B* and *C*). Mutating the residue at this site to alanine or other residues with opposite hydrophobicity/hydrophilicity in *Pp*OngB, *Af*Dam, and *Bb*Dam resulted in marked reduction or loss of enzymatic activities (Figs. 3E, 6, *D* and *E*), demonstrating the key role of this residue in determining substrate specificities of GlcNAc1A deacetylases and D-aminoacylases. Another key residue, Ser292, stabilizes the C3-hydroxy group of GlcNAc1A. This residue is replaced by threonine conserved in *Af*Dam-like and *Bb*Dam-like sequences (Fig. 6, *B* and *C*), where its additional methyl group contributes hydrophobicity and impacts substrate recognition. Mutation of Ser292 to threonine in *Pp*OngB significantly reduced its enzymatic activity and catalytic efficiency (*k*_cat_/*K*_m_), and conversely in *Af*Dam and *Bb*Dam, Thr mutations had similar effects (Figs. 3*E*, 6, *D* and *E*, and Table 1). Both Ser292 and Gln298 and their counterparts are positioned in/near the loop 2 of the insertion domains of *Pp*OngB and D-aminoacylases, emphasizing the role of this region in substrate recognition.

**Figure 7:**
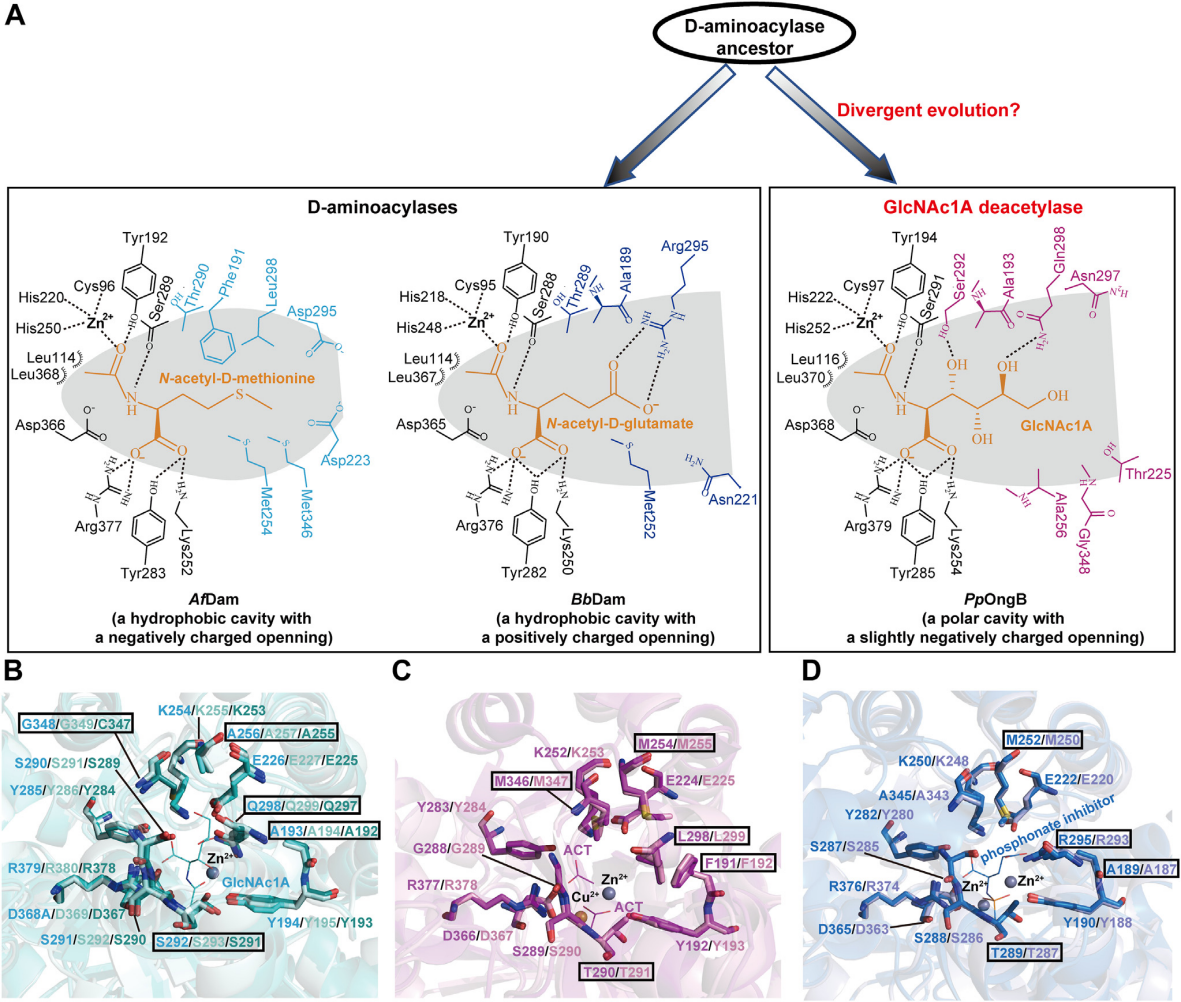
**The proposed molecular mechanism driving the formation of the carbohydrate deacetylase activity of *Pp*OngB and its homologs from D-aminoacylases.***A*, a proposed mechanism underlying the evolution of *Pp*OngB. Structural modifications in the catalytic cavity of an ancestral D-aminoacylase, especially in electrostatics, are proposed to drive the evolution of *Pp*OngB into a GlcNAc1A deacetylase. The bound substrates in *Pp*OngB and D-aminoacylases are highlighted in *orange*. Key residue changes in the catalytic cavities of D-aminoacylases and *Pp*OngB, which lead to their different electrostatic properties, are indicated in *cyan* (*Af*Dam), *slate* (*Bb*Dam), and *magenta* (*Pp*OngB), respectively. Interactions between *Af*Dam and *N*-acetyl-D-methionine are shown based on molecular docking analysis. Possible hydrogen bonds are represented by *dashed lines*. *B*, superposition of the active sites of *Pp*OngB (*cyan*), *Pa*OngB (*pale cyan*), and *Pf*OngB (*deep teal*) suggesting that *Pp*OngB-like enzymes have similarly polar catalytic cavities. The bound GlcNAc1A in *Pp*OngB is shown as *cyan lines*. *C*, superposition of the active sites of *Af*Dam (*magenta*) and its homolog, *Ax*Dam (*pink*), suggesting that *Af*Dam-like enzymes have similarly hydrophobic catalytic cavities. The two acetate molecules bound in *Af*Dam are shown as *magenta lines*. *D*, superposition of the active sites of *Bb*Dam (*slate*) and its homolog, D-AGase (*light blue*), suggesting that *Bb*Dam-like enzymes have similar catalytic cavities with positively charged openings. The bound phosphate inhibitor in *Bb*Dam is shown as *slate lines*. In (*B*)–(*D*), the structures of two *Pp*OngB homologs (*Pa*OngB and *Pf*OngB), an *Af*Dam homolog (*Ax*Dam) and a *Bb*Dam homolog (D-AGase) are predicted using AlphaFold3, highlighting residues associated with their differently charged catalytic cavities in *boxes*. ACT, acetate; GlcNAc1A, 2-(acetylamino)-2-deoxy-D-gluconic acid.

Based on the phylogenetic and structural analyses above, we propose that GlcNAc1A deacetylases might share a common D-aminoacylase ancestor with D-aminoacylases such as *Af*Dam and *Bb*Dam and underwent a divergent evolution. The ancestral D-aminoacylase adopted structural modifications in its catalytic cavity, especially in electrostatics, to evolve into a GlcNAc1A deacetylase (Fig. 7*A*). Further analysis of the structures of functional homologs to *Pp*OngB, *Af*Dam, and *Bb*Dam, predicted by AlphaFold3 (49), indicated that structural modifications in the catalytic cavities drive the functional shift between GlcNAc1A deacetylases and D-aminoacylases (Fig. 7, *B*–*D*). The affinities of *Pp*OngB and its homologs for GlcNAc1A are 1 to 2 orders of magnitude lower than those of *Af*Dam (45) and *Bb*Dam (41) for *N*-acetyl-D-amino acids (Table 1), also supporting the hypothesis that GlcNAc1A deacetylases evolved later from D-aminoacylases. *Pp*OngB-like sequences are abundant in marine Gammaproteobacteria and also found in terrestrial Gammaproteobacteria (18), suggesting that the emergence of GlcNAc1A deacetylases from D-aminoacylases is important for oxidative chitin degradation. Our study on GlcNAc1A deacetylases offers valuable insights into the evolution of the GlcNAc1A catabolic pathway, enhancing our understanding of oxidative chitin degradation.

## Conclusions

Our biochemical characterization of *Pp*OngB and its homologs confirms their specificity as GlcNAc1A deacetylases. Structural and mutational analyses identify essential residues involved in substrate binding and catalysis within *Pp*OngB. This enzyme utilizes a D-aminoacylase-like (β/α)_8_-barrel fold for GlcNAc1A deacetylation in a metal-dependent manner. It is a β-mononuclear amidohydrolase with a subtype V metal-binding site. Phylogenetic and structural comparisons position *Pp*OngB and its homologs as a new family of carbohydrate de-*N*-acetylases. The evolutionary adaptation of *Pp*OngB likely stems from structural modifications in the catalytic cavity of an ancestral D-aminoacylase, particularly in electrostatic properties, allowing *Pp*OngB to specialize as a GlcNAc1A deacetylase. Our study on GlcNAc1A deacetylases provides new insights into the mechanism of chitin catabolism *via* oxidative degradation. This study also establishes a connection between amino sugar catabolism and amino acid catabolism, shedding light on the evolution of oxidative chitin degradation, especially for the GlcNAc1A catabolism.

## Experimental procedures

### Gene cloning and mutagenesis

The *PpongB* gene was amplified from the genomic DNA of the marine bacterium *P. prydzensis* ACAM 620 and cloned into the vector pET-22b, which includes a His tag. Using the plasmid pET22b-*PpongB* as a template, site-directed mutations in *Pp*OngB (Table S2) were introduced by a modified QuikChange site-directed mutagenesis method (50). Similarly, two homologs of *PpongB*, *PaongB* (GenBank accession no. WP_209438165), and *PfongB* (GenBank accession no. WP_039496229) from marine bacteria *P. arabiensis* and *P. flavipulchra* DSM 14401, respectively, were cloned. Additionally, we synthesized two D-aminoacylase-encoding genes, *Afdam* (40) and *Bbdam* (41), and constructed their mutants. All primers used in this study are listed in Table S2. All recombinant plasmids were verified by sequencing.

### Protein expression and purification

Proteins including *Pp*OngB, *Pa*OngB, *Pf*OngB, *Af*Dam, *Bb*Dam, and their mutants were expressed in *Escherichia coli* BL21 (DE3). Cells were cultured at 37 °C until the absorbance at 600 nm reached 0.6 and then induced by the addition of 0.2 mM IPTG at 16 °C for 20 h. Cells were collected and disrupted using the JN-02C French press (JNBIO) in 50 mM Tris–HCl buffer (pH 8.0) containing 100 mM NaCl and 5 mM imidazole. The recombinant proteins were initially purified by nickel affinity chromatography (QIAGEN) and subsequently fractionated by anion exchange on a SOURCE 15Q column (GE HealthCare). The target protein was then collected using gel filtration on a Superdex G-200 column (GE HealthCare). Conalbumin (75 kDa) and ovalbumin (43 kDa) were used as protein size standards. Protein concentration was determined using the Pierce BCA Protein Assay Kit (Thermo Fisher Scientific), and the purified proteins were stored at −80 °C.

### ICP-MS analysis

Purified *Pp*OngB was desalted into metal-free buffer (10 mM Tris–HCl, 100 mM NaCl, pH 8.0). It was then digested in concentrated nitric acid to a final concentration of 10%. After filtering the sample, it was analyzed using ICP-MS (PerkinElmer). In addition to the standard calibrating solutions, buffer-spiked standards were used to minimize any interference from the buffer during metal analysis.

### Biochemical characterization

The standard reaction system (50 μl) contained 10 mM Bis-Tris buffer (pH 7.5), 10 mM GlcNAc1A (Aladdin), and an appropriate concentration of enzyme. Reactions were incubated at 30 °C for 15 min and terminated by boiling for 15 min. The resulting primary amine products were measured using the ninhydrin reagent (51). Briefly, 20 μl of the reaction mixture was mixed with 100 μl of the ninhydrin-sodium citrate reagent and boiled for 20 min. After adding 500 μl of a 50% *n*-propanol solution, the reaction product was detected at 600 nm. One unit of enzyme activity (U) is defined as the amount of enzyme required to release 1 μmol of primary amine per min.

The optimum temperature for *Pp*OngB activity was determined ranging from 10 to 60 °C at pH 7.5. For the thermostability assay, the enzyme was preincubated at 30 °C, 35 °C, and 40 °C for different time intervals, and the residual activity was measured at pH 7.5 and 30 °C. The optimum pH for *Pp*OngB activity was determined ranging from pH 5.0 to 10.0 with Britton–Robinson buffer at 30 °C. Substrate specificity assays of *Pp*OngB, *Pa*OngB, *Pf*OngB, *Af*Dam, and *Bb*Dam were performed using various substrates, including amino sugars GlcNAc1A, GlcNAc, GalNAc, ManNAc, MurNAc, Neu5Ac, GlcNAc-GlcNAc1A, and (GlcNAc)_2_ as well as *N*-acetyl-D-amino acids such as *N*-acetyl-D-arginine, *N*-acetyl-D-glutamate, *N*-acetyl-D-serine, *N*-acetyl-D-alanine, *N*-acetyl-D-leucine, *N*-acetyl-D-methionine, *N*-acetyl-D-phenylalanine, *N*-acetyl-D-proline, *N*-acetyl-D-tryptophan, and *N*-acetyl-D-valine.

Enzyme kinetic assays of *Pp*OngB, *Pa*OngB, *Pf*OngB, and *Pp*OngB mutants were performed in 10 mM Bis-Tris buffer (pH 7.5) at 30 °C using GlcNAc1A as substrate over a concentration range of 2 to 120 mM. Kinetic parameters were calculated through nonlinear regression fitted directly to the Michaelis–Menten equation using Origin 9.0 software. The overall secondary structures of WT *Pp*OngB and its mutants were studied at 25 °C using a J-810 CD spectropolarimeter (JASCO). CD spectra were collected from 200 to 250 nm at a scanning rate of 200 nm/min with a path length of 0.1 cm. The protein concentrations for CD spectroscopy assays were set at 0.1 mg/ml.

### Preparation of the apo enzyme and its metal reconstruction

Trace metals in 10 mM Bis-Tris buffer (pH 7.5) were removed by a Chelex 100 chelating ion exchange resin (Bio-Rad) to prepare the metal-free buffer. To prepare the metal-free *Pp*OngB, the WT enzyme (0.4 mg/ml) was dialyzed against 10 mM DPA in 10 mM metal-free Bis-Tris buffer (pH 7.5) using a 3.3 kDa molecular weight cutoff dialysis cassette, with three changes of the dialysis buffer over 2 days at 4 °C. The apo *Pp*OngB was then desalted using a PD-10 desalting column (GE HealthCare) equilibrated with 10 mM metal-free Bis-Tris buffer (pH 7.5) to remove DPA.

To study the effect of different metal ions on *Pp*OngB activity, the apo enzyme (1.5 μM) was incubated with 5 μM metal ions for 0.5 h at 4 °C, and its activity was determined with 10 mM GlcNAc1A substrate in 10 mM metal-free Bis-Tris buffer (pH 7.5) at 30 °C. For the metal reconstitution study, aliquots of the apo enzyme (1.5 μM) were incubated with 0 to 6 molar equivalents of ZnCl_2_ for 20 h at 4 °C. The enzymatic activity of the reconstituted protein was determined with 10 mM GlcNAc1A substrate in 10 mM metal-free Bis-Tris buffer (pH 7.5) at 30 °C.

### Crystallization, data collection, and structure determination

The protein concentrations of *Pp*OngB and its inactive mutant D368A for crystallization were 8.0 mg/ml in 10 mM Tris–HCl (pH 8.0) containing 100 mM NaCl. To obtain the crystals of the D368A mutant in complex with GlcNAc1A (the *Pp*OngB–GlcNAc1A complex), D368A mutant was mixed with GlcNAc1A at a molar ratio of 1: 50 and then incubated at 4 °C for 4 h before crystallization. The crystals of the WT *Pp*OngB and the *Pp*OngB-GlcNAc1A complex suitable for X-ray diffraction were obtained at 18 °C after 1 week in the buffer containing 0.5 M ammonium sulfate, 0.1 M sodium citrate tribasic dihydrate (pH 6.0), and 1.0 M lithium sulfate monohydrate by the hanging-drop vapor diffusion method. All the X-ray diffraction data of crystals were collected on the BL19U1 beamline at the Shanghai Synchrotron Radiation Facility using the PILATUS3 6M detector (52). Processing and scaling of raw data were performed using the HKL3000 program (53). The crystal structure of *Pp*OngB was solved by molecular replacement using Phenix (54), with its modeled structure predicted by AlphaFold2 (55) as the staring model. Subsequent refinement was performed using Phenix (54) and Coot (56) alternately. The structure of the *Pp*OngB-GlcNAc1A complex was determined using the crystal structure of *Pp*OngB as the staring model. The qualities of the final models are summarized in Table S1. All the structure figures were processed using PyMOL program.

### Protein structure prediction

Based on blasting analysis, the D-aminoacylase *Ax*Dam from *Achromobacter xylosoxidans* (formerly *Alcaligenes xylosoxydans* subsp. *xylosoxydans* A-6) (57) was identified as a homolog of *Af*Dam with 86% sequence identity, which could deacetylate *N*-acetyl-D-methionine. Another D-aminoacylase, D-AGase, from *A*. *xylosoxidans* (58) was recognized as a homolog of *Bb*Dam with 80% sequence identity. D-AGase was characterized as a functional *N*-acyl-D-glutamate amidohydrolase (58). The structures of *Pa*OngB, *Pf*OngB, *Ax*Dam, and D-AGase were predicted using AlphaFold3 (49) and compared to analyze the catalytic cavities of *Pp*OngB-like, *Af*Dam-like and *Bb*Dam-like enzymes. In addition, Molegro Virtual Docker 6.0 (59) was used to conduct *Af*Dam (PDB code 1M7J) and *N*-acetyl-D-methionine docking. The default docking parameters were adopted except that the number of runs, the max iterations, and the population size in each run were set to 50, 3000, and 100, respectively. Cluster analysis was performed on different poses with a RMSD threshold of 1.0 Å. The conformation with the lowest estimated binding energy was selected as the best probable binding mode.

### Phylogenetic analysis

Characterized carbohydrate de-*N*-acetylases and their homologs from CE families 4, 9, 11, 14, and 18 were retrieved from the CAZy database. For each family, the source strains of selected protein sequences represented 6 to 14 of the most abundant bacterial/eukaryotic groups within this family at the genus level, with sequence identities within each family ranging from ≤27% to ≥88%. *Af*Dam-like and *Bb*Dam-like D-aminoacylases, sharing intragroup sequence identities between 50% and 96%, were downloaded from the NCBI nr database. Based on the distribution of *Pp*OngB in marine and terrestrial bacteria (18), *Pp*OngB-like sequences were obtained from the NCBI nr database, sharing sequence identities between 50% and 99%. The selected D-aminoacylases and *Pp*OngB-like sequences covered at least six of the most abundant bacterial genera containing *Af*Dam-like, *Bb*Dam-like, or *Pp*OngB-like sequences. To reveal the relationship between *Pp*OngB-like sequences and other carbohydrate de-*N*-acetylases as well as D-aminoacylases, a total of 150 selected protein sequences were aligned using MAFFT v7.471 (60) with the -auto option, and trimmed using trimAl v1.4 (61) with the -gappyout option. Maximum likelihood trees were constructed using IQ-TREE v2.1.2 (parameters: -m LG + F + R10 -bb 1000 -bnni -seed 500) (62, 63) and RAxML v8.2.4 (parameters: raxmlHPC-PTHREADS-AVX -m PROTGAMMAAUTO -p 12,345 -x 12345 -N autoMRE) (64, 65), respectively. The resulting phylogenetic trees were visualized using the Interactive Tree of Life (iTOL) v5 (66).

### Statistical analysis

Statistical analyses were carried out using Statistical Product and Service Solutions (SPSS) 29.0. An independent two-sample *t* test was performed for pairwise comparisons.

## Data availability

Atomic coordinates and structure factors of the wild-type *Pp*OngB and the *Pp*OngB-GlcNAc1A complex have been deposited in Protein Data Bank (PDB) under the accession numbers 9KB1 and 9KB3, respectively.

## Supporting information

This article contains supporting information.

## Conflict of interest

The authors declare that they have no conflicts of interest with the contents of this article.
