# Supplementary material for: Carbohydrate deacetylase, a key enzyme in oxidative chitin degradation, is evolutionarily linked to amino acid deacetylase[image]
Source: J Biol Chem. 2025 Mar 18;301(4):108420. doi: 10.1016/j.jbc.2025.108420 (PMC12017989; doi:10.1016/j.jbc.2025.108420)
Supplement: Supporting Information [file mmc1.docx]

Supporting Information

**Carbohydrate deacetylase, a key enzyme in oxidative chitin degradation, is evolutionarily linked to amino acid deacetylase**

Jing-Ping Wang^1^, Xiang-Ming Zhao^1^, Xiao-Lei Liu^1^, Wen-Xin Jiang^1^, Chao Gao^1^, Hai-Yan Cao^2.3^, Hai-Tao Ding^4^, Qi-Long Qin^1^, Xiu-Lan Chen^1^, Yu-Zhong Zhang^2,3,5^*, Ping-Yi Li^1^*

^1^State Key Laboratory of Microbial Technology, Shandong University, Qingdao, 266237, China.

^2^MOE Key Laboratory of Evolution and Marine Biodiversity, Frontiers Science Center for Deep Ocean Multispheres and Earth System & College of Marine Life Sciences, Ocean University of China, Qingdao, 266003, China.

^3^Laboratory for Marine Biology and Biotechnology, Qingdao Marine Science and Technology Center & Laoshan Laboratory, Qingdao, 266237, China.

^4^Antarctic Great Wall Ecology National Observation and Research Station, Polar Research Institute of China, Ministry of Natural Resources, Shanghai, 200136, China.

^5^Marine Biotechnology Research Center, State Key Laboratory of Microbial Technology, Shandong University, Qingdao, 266237, China.

*Corresponding authors: zhangyz@sdu.edu.cn (Y.-Z.Z.); lipingyipeace@sdu.edu.cn (P.-Y.L.)

**Running title:** Catalysis and evolution of a novel carbohydrate esterase

**Keywords**: carbohydrate deacetylase, oxidative chitin degradation, catalysis, structural adaption, evolution

**Supplementary** **Tables**

**Table S1** **Crystallographic data collection and refinement of *Pp*OngB.**

| **Parameters** | ***Pp*OngB** | ***Pp*OngB-GlcNAc1A** |
| --- | --- | --- |
| Diffraction data |  |  |
| Space group | P3_1_21 | P3_1_21 |
| Unit cell |  |  |
| a, b, c (Å) | 87.6  87.6  143.3 | 86.8  86.8  141.6 |
| α, β, γ (°) | 90.0  90.0  120.0 | 90.0  90.0  120.0 |
| Resolution range (Å) | 75.86-1.54  (1.66-1.54)*^a^* | 66.41-2.29  (2.29-2.42) |
| *R*_merge_^b^ | 0.126 | 0.314 |
| CC1/2 | 0.999 | 0.995 |
| I/Sigma | 14.7 | 9.4 |
| Completeness (%) | 100 | 99.5 |
| Refinement statistics |  |  |
| Resolution range | 32.40-1.77  (1.83-1.77) | 51.55-2.29  (2.37-2.29) |
| *R*_work_ (%) | 17.24 | 17.2 |
| *R*_free_ (%) | 19.9 | 21.9 |
| RMS (bonds) | 0.006 | 0.008 |
| RMS (angles) | 0.84 | 0.93 |
| Wilson *B*-factor (Å^2^) | 24.74 | 29.89 |
| Clashscore | 0.98 | 4.43 |
| Average B-factor (Å^2^) | 30.73 | 29.90 |
| Macromolecules/solvent/ligands | 29.05/43.02/32.53 | 28.70/32.57/30.59 |
| Ramachandran plot (%) |  |  |
| Favored | 97.68 | 97.27 |
| Allowed | 2.32 | 2.52 |
| Outliers | 0 | 0.21 |
| Mutation | No mutation | D368A |
| PDB identifier | 9KB1 | 9KB3 |

*^a^*Numbers in parentheses refer to data in the highest resolution shell.

^b^*R*_merge_ = Σ_hkl_Σ_i_|*I*(*hkl*)_i_ - <*I*(*hkl*)>|/Σ_hkl_Σ_i_ < *I*(*hkl*)_i_>.

**Table S2** **Primers used in this study.**

| **Enzyme** | **Primer** | **Sequence (5’→3’)** |
| --- | --- | --- |
| *Pp*OngB | *Pp*OngB_F | AAGAAGGAGATATACATATGATGCAGTACGATATCTCGCAACCAG |
|  | *Pp*OngB_R | TGGTGGTGGTGGTGCTCGAGATCATGTTTACTTGCTCCTAAGGATGTTAAAAA |
|  | H68A_F | AAATCATCGTGAGTAGCTACATCAATAAAGCCTGGCGCTAAACAC |
|  | H68A_R | GTGTTTAGCGCCAGGCTTTATTGATGTAGCTACTCACGATGATTT |
|  | H70A_F | TACTTCTAAATCATCGGCAGTATGTACATCAATAAAGCCTGGCGC |
|  | H70A_R | GCGCCAGGCTTTATTGATGTACATACTGCCGATGATTTAGAAGTA |
|  | C97A_F | CTGCACTAATGCCAGCATTTCCTGTAATAACGGTAGTCACTCC |
|  | C97A_R | GGAGTGACTACCGTTATTACAGGAAATGCTGGCATTAGTGCAG |
|  | Y194A_F | CGCCTGATTAGCATTTTTAGCGGCAAGGCCAGTACTCAAG |
|  | Y194A_R | CTTGAGTACTGGCCTTGCCGCTAAAAATGCTAATCAGGCG |
|  | H222A_F | GTCAAACTCGGTGCGTAAAGCTGTGGTATACAGCGCATCG |
|  | H222A_R | CGATGCGCTGTATACCACAGCTTTACGCACCGAGTTTGAC |
|  | E226A_F | CAAGTACTGCGTCAAACGCGGTGCGTAAATGTGTG |
|  | E226A_R | CACACATTTACGCACCGCGTTTGACGCAGTACTTG |
|  | H252A_F | GTTATTTTTACCTGCGCATTTAAGGGCCGAAATAATCACCTTAATATCGAAT |
|  | H252A_R | ATTCGATATTAAGGTGATTATTTCGGCCCTTAAATGCGCAGGTAAAAATAAC |
|  | K254A_F | TTATTTTTACCTGCGCATGCAAGGTGCGAAATAATCACCTTAATATCGAATGC |
|  | K254A_R | GCATTCGATATTAAGGTGATTATTTCGCACCTTGCATGCGCAGGTAAAAATAA |
|  | Y285A_F | TGCGGCATACGGGGCAGCGTCACAACTGCATTTTGAATGC |
|  | Y285A_R | GCATTCAAAATGCAGTTGTGACGCTGCCCCGTATGCCGCA |
|  | S290A_F | AAATCTAAGGTGCTGGAAGCTGCGGCATACGGGTAAGC |
|  | S290A_R | GCTTACCCGTATGCCGCAGCTTCCAGCACCTTAGATTT |
|  | S291A_F | TAAGGTGCTGGCACTTGCGGCATACGGGTAAG |
|  | S291A_R | CTTACCCGTATGCCGCAAGTGCCAGCACCTTA |
|  | S292A_F | CTTGGTTTAAATCTAAGGTGGCGGAACTTGCGGCATACGGG |
|  | S292A_R | CCCGTATGCCGCAAGTTCCGCCACCTTAGATTTAAACCAAG |
|  | S292T_F | TGGTTTAAATCTAAGGTGGTGGAACTTGCGGCATAC |
|  | S292T_R | GTATGCCGCAAGTTCCACCACCTTAGATTTAAACCA |
|  | Q298A_F | ATCAAAATCATCGGTCACTGCGTTTAAATCTAAGGTGCTGGAACTTG |
|  | Q298A_R | CAAGTTCCAGCACCTTAGATTTAAACGCAGTGACCGATGATTTTGAT |
|  | Q298L_F | ATCAAAATCATCGGTCACTAGGTTTAAATCTAAGGTGCTGG |
|  | Q298L_R | CCAGCACCTTAGATTTAAACCTAGTGACCGATGATTTTGAT |
|  | Q298N_F | AATATCAAAATCATCGGTCACATTGTTTAAATCTAAGGTGCTGGAACTTGC |
|  | Q298N_R | GCAAGTTCCAGCACCTTAGATTTAAACAATGTGACCGATGATTTTGATATT |
|  | Q298R_F | TATCAAAATCATCGGTCACTCTGTTTAAATCTAAGGTGCTGGAACTTGC |
|  | Q298R_R | GCAAGTTCCAGCACCTTAGATTTAAACAGAGTGACCGATGATTTTGATA |
|  | D368A_F | CGCACGGTAAGCCGGCGGAGCCAATCATG |
|  | D368A_R | CATGATTGGCTCCGCCGGCTTACCGTGCG |
|  | R379A_F | AATGAGCCCCATAAAGCCGGATGCGGATGGGG |
|  | R379A_R | CCCCATCCGCATCCGGCTTTATGGGGCTCATT |
| *Pa*OngB | *Pa*OngB_F | TAAGAAGGAGATATACATATGATGATGCACAGCAAAGACT |
|  | *Pa*OngB_R | GTGGTGGTGGTGCTCGAGCTCTCCAGTTGAAGTTAAAAA |
| *Pf*OngB | *Pf*OngB_F | TTTAAGAAGGAGATATACATATGATGCTGAGCTCATC |
|  | *Pf*OngB_R | GTGGTGGTGGTGCTCGAGGTTTTGACGCGTTA |
| *Af*Dam | T290A_F | CTGTTTCAGCATGGCGCTGCCCGCCACAT |
|  | T290A_R | ATGTGGCGGGCAGCGCCATGCTGAAACAG |
|  | T290S_F | CCTGTTTCAGCATGCTGCTGCCCGCCAC |
|  | T290S_R | GTGGCGGGCAGCAGCATGCTGAAACAGG |
|  | L298A_F | CGGCCCGCCAGCGCCACGCGATCCTG |
|  | L298A_R | CAGGATCGCGTGGCGCTGGCGGGCCG |
|  | L298I_F | GTACGGCCCGCCAGTATCACGCGATCCTGT |
|  | L298I_R | AACAGGATCGCGTGATACTGGCGGGCCGTAC |
|  | L298Q_F | GGCCCGCCAGCTGCACGCGATCC |
|  | L298Q_R | GGATCGCGTGCAGCTGGCGGGCC |
| *Bb*Dam | T289A_F | CGGAATCAGAATGGCGCTGCTGCCCGGA |
|  | T289A_R | TCCGGGCAGCAGCGCCATTCTGATTCCG |
|  | T289S_F | CGGAATCAGAATGCTGCTGCTGCCCGG |
|  | T289S_R | CCGGGCAGCAGCAGCATTCTGATTCCG |
|  | R295A_F | TGGTTTCCGCGGCTTCCGGAATCAGAATGGTGC |
|  | R295A_R | GCACCATTCTGATTCCGGAAGCCGCGGAAACCA |
|  | R295K_F | ATCAATGGTTTCCGCCTTTTCCGGAATCAGAATGGTGCTGCTGC |
|  | R295K_R | GCAGCAGCACCATTCTGATTCCGGAAAAGGCGGAAACCATTGAT |
|  | R295L_F | CATCAATGGTTTCCGCGAGTTCCGGAATCAGAATG |
|  | R295L_R | CATTCTGATTCCGGAACTCGCGGAAACCATTGATG |
|  | R295Q_F | TATCATCAATGGTTTCCGCCTGTTCCGGAATCAGAATGGT |
|  | R295Q_R | ACCATTCTGATTCCGGAACAGGCGGAAACCATTGATGATA |

**Supplementary Figures**


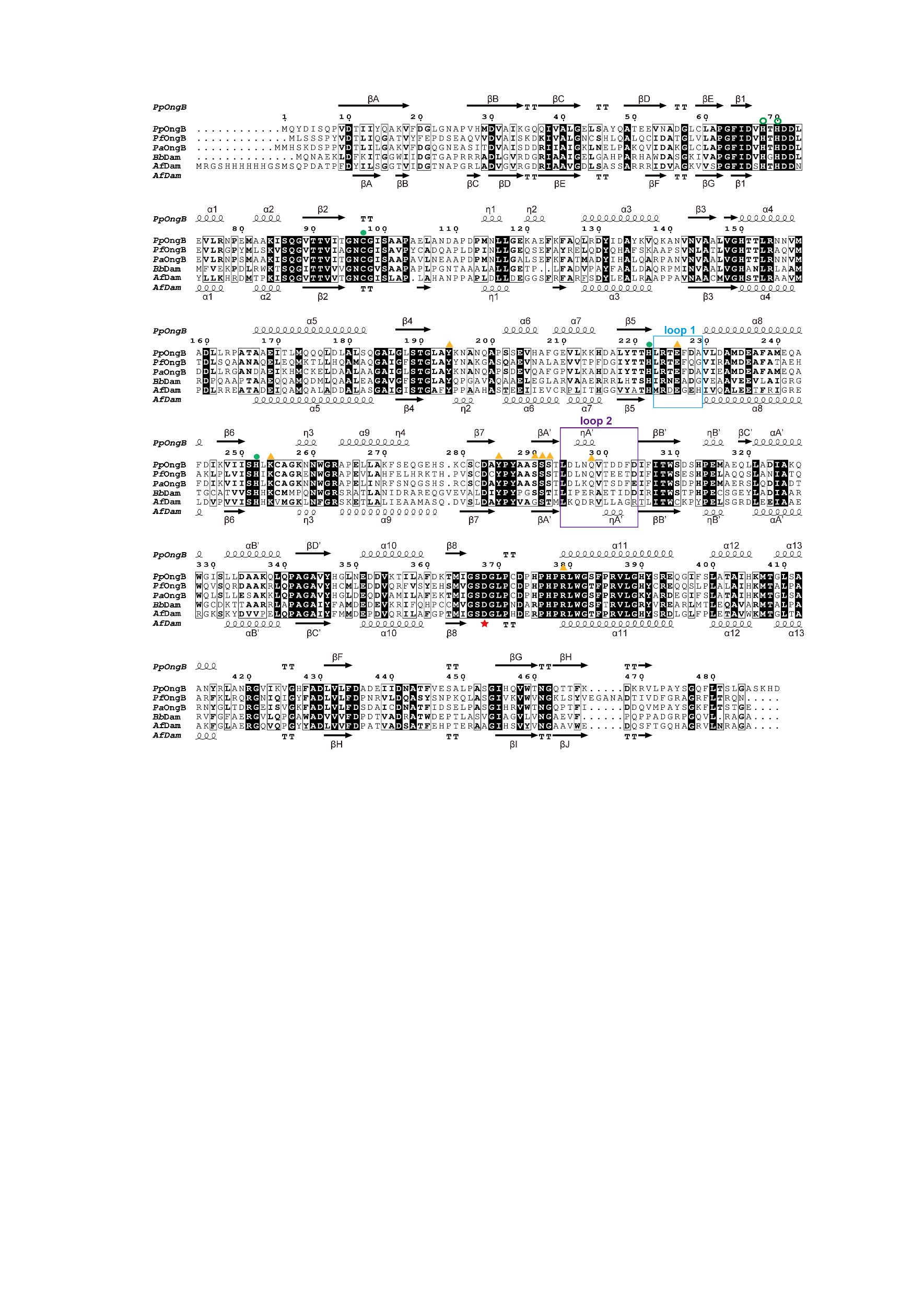


**Figure S1 Sequence alignment of *Pp*OngB and its homologs and D-aminoacylases.** A red star indicates the potential catalytic Asp residue conserved in *Pp*OngB and its homologs and D-aminoacylases. Green solid circles indicate potential metal binding residues conserved in *Pp*OngB and its homologs and D-aminoacylases. Green open circles indicate residues that can accommodate a second metal ion in some cases but is not required for catalysis. Key residues of *Pp*OngB involved in the binding of the substrate GlcNAc1A are marked by orange triangles. Loops 1 and 2 of *Pp*OngB and D-aminoacylases are boxed with different colors.


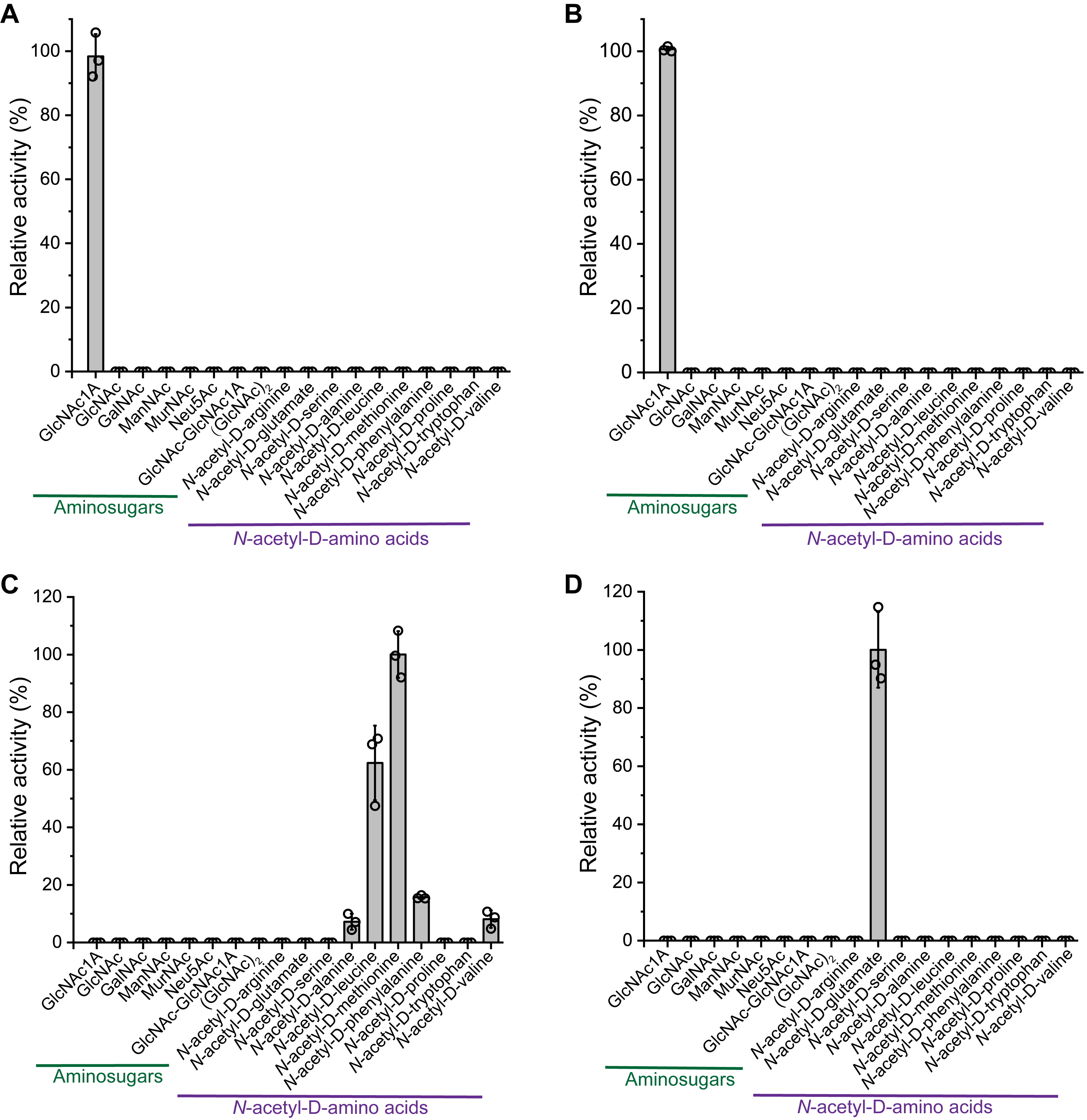


**Figure S2 Substrate specificity analyses of** ***Pa*OngB, *Pf*OngB, *Af*Dam and *Bb*Dam.** (A) Substrate specificity analysis of *Pa*OngB at 30℃ in 10 mM Bis-Tris buffer (pH 7.5) using10 mM substrate and 0.9 µM enzyme. The specific activity of *Pa*OngB (4.4 U/mg) against GlcNAc1A was taken as 100%. (B) Substrate specificity analysis of *Pf*OngB at 30℃ in 10 mM Bis-Tris buffer (pH 7.5) using10 mM substrate and 0.7 µM enzyme. The specific activity of *Pf*OngB (5.0 U/mg) against GlcNAc1A was taken as 100%. (C) Substrate specificity analysis of *Af*Dam at 40℃ in 10 mM Bis-Tris buffer (pH 7.0) using 10 mM substrate and 0.2 µM enzyme. The specific activity of *Af*Dam against *N*-acetyl-D-methionine (31.1 U/mg) was taken as 100%. (D) Substrate specificity analysis of *Bb*Dam at 30℃ in 50 mM HEPES buffer (pH 8.0) using 10 mM substrate and 0.8 µM enzyme. The specific activity of *Bb*Dam against *N*-acetyl-D-glutamate (7.7 U/mg) was taken as 100%. The data shown in (A)-(D) are from triplicate experiments (mean ± S.D.). GalNAc, *N*-acetyl-D-galactosamine; ManNAc, *N*-acetyl-D-mannosamine; MurNAc, *N*-acetylmuramic acid; Neu5Ac, *N*-acetylneuraminic acid.


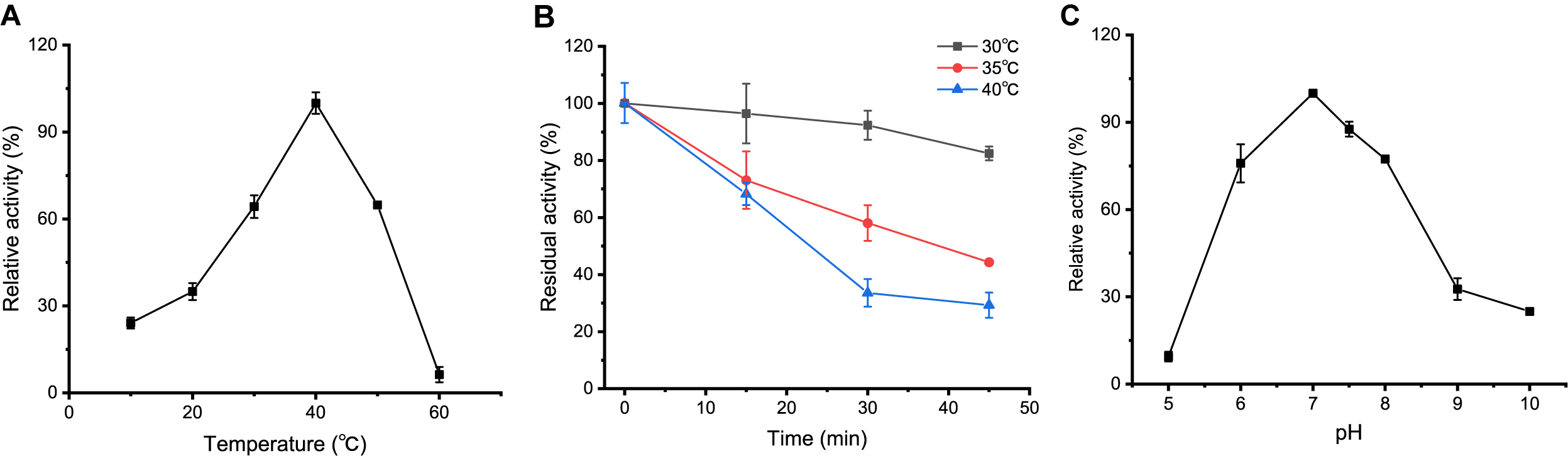


**Figure S3 Biochemical characterization of *Pp*OngB**. (A) Effect of temperature on the activity of *Pp*OngB. Assays were performed in 10 mM Bis-Tris buffer (pH 7.5) with 10 mM GlcNAc1A and 0.8 µM enzyme at temperatures ranging from 10℃ to 60℃. The highest activity of *Pp*OngB at 40℃ was taken as 100%. (B) Effect of temperature on the stability of *Pp*OngB. The enzyme was incubated at 30℃, 35℃ and 40℃ for different time intervals, and the residual activity was measured at pH 7.5 and 30℃. (C) Effect of pH on the activity of *Pp*OngB. Assays were conducted in Britton-Robinson buffer (pH 5.0-10.0) with 10 mM GlcNAc1A and 0.8 µM enzyme at 30℃. The highest activity of *Pp*OngB at pH 7.0 was taken as 100%. The data shown in (A)**-**(C) are from triplicate experiments (mean ± S.D.).


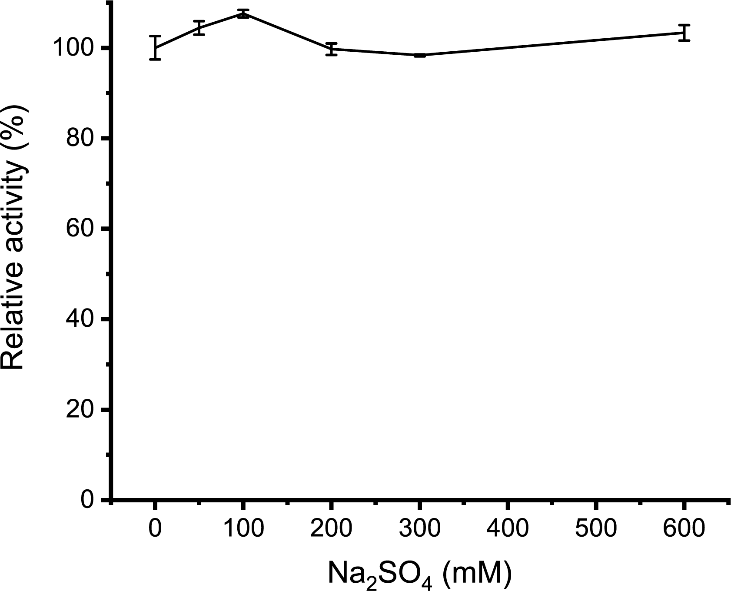


**Figure S4 Effect of Na_2_SO_4_ on the activity of *Pp*OngB.** The wild-type *Pp*OngB (0.9 µM) was incubated with varying concentrations of Na_2_SO_4_ at 4℃ for 20 min and then the activity was measured against 10 mM GlcNAc1A in 10 mM Bis-Tris buffer (pH 7.5) at 30℃. The activity of *Pp*OngB in 0 M sodium sulfate was taken as 100%. The data shown are from triplicate experiments (mean ± S.D.).

**
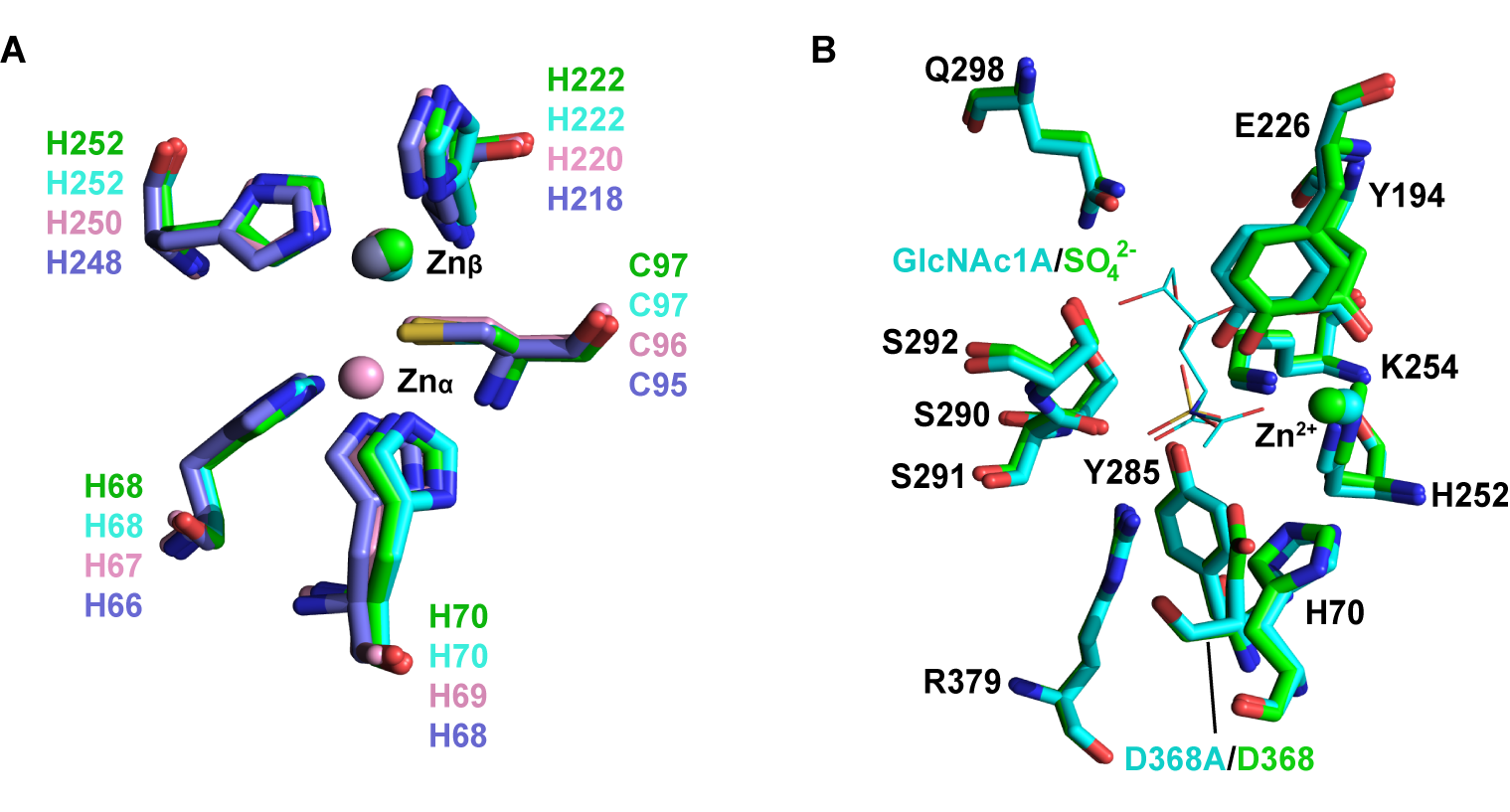
**

**Figure S5 Superimposition of the active sites of the wild-type *Pp*OngB and the *Pp*OngB-GlcNAc1A complex (and D-aminoacylases).** (A) Superimposition of the metal-binding ligands in the wild-type *Pp*OngB (green), the *Pp*OngB-GlcNAc1A complex (cyan), *Af*Dam (pink) and *Bb*Dam (slate). (B) Superimposition of the substrate-binding residues of the wild-type *Pp*OngB (green) and the *Pp*OngB-GlcNAc1A complex (cyan). The bound GlcNAc1A in mutant D368A is shown as cyan lines.


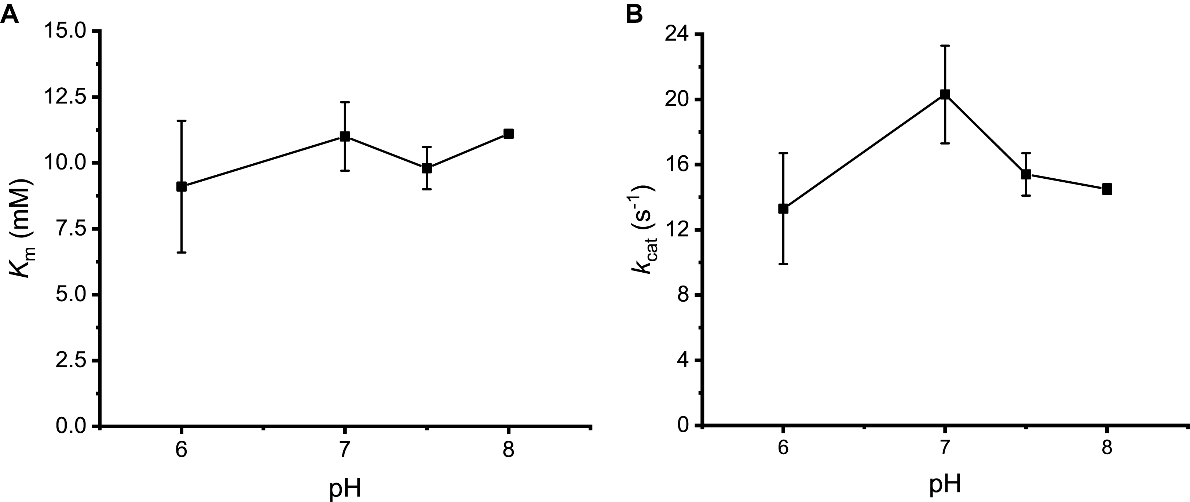


**Figure S6 Effect** **of pH on the *K*_m_ (A) and *k*_cat_ (B) of *Pp*OngB.** Reactions were conducted in 10 mM Bis-Tris buffer with different pH values at 30℃, using GlcNAc1A as the substrate over a concentration range of 0-30 mM and 0.25 µM enzyme. The data shown in (A) and (B) are from triplicate experiments (mean ± S.D.).


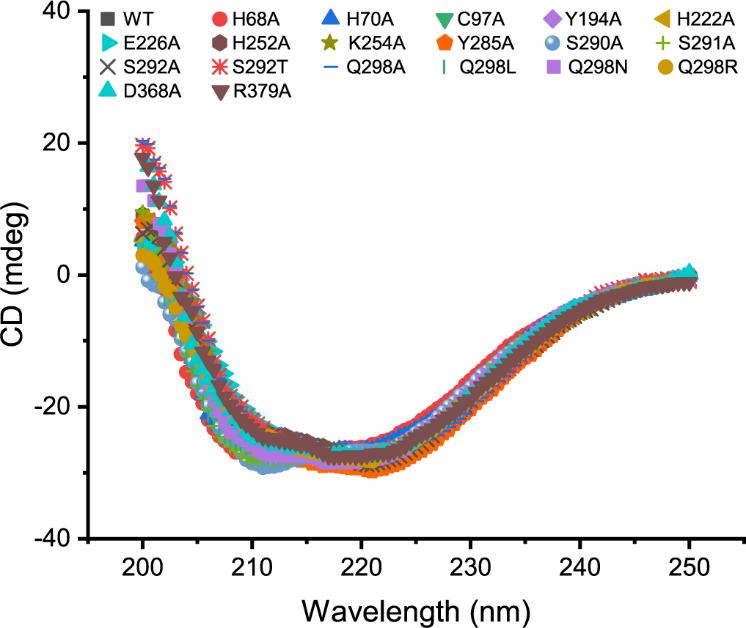


**Figure S7 CD spectra of wild-type *Pp*OngB and its mutants.** CD spectra were collected from 200 to 250 nm at a scanning rate of 200 nm/min with a path length of 0.1 cm. The protein concentrations for CD spectroscopy assays were set at 0.1 mg/ml.


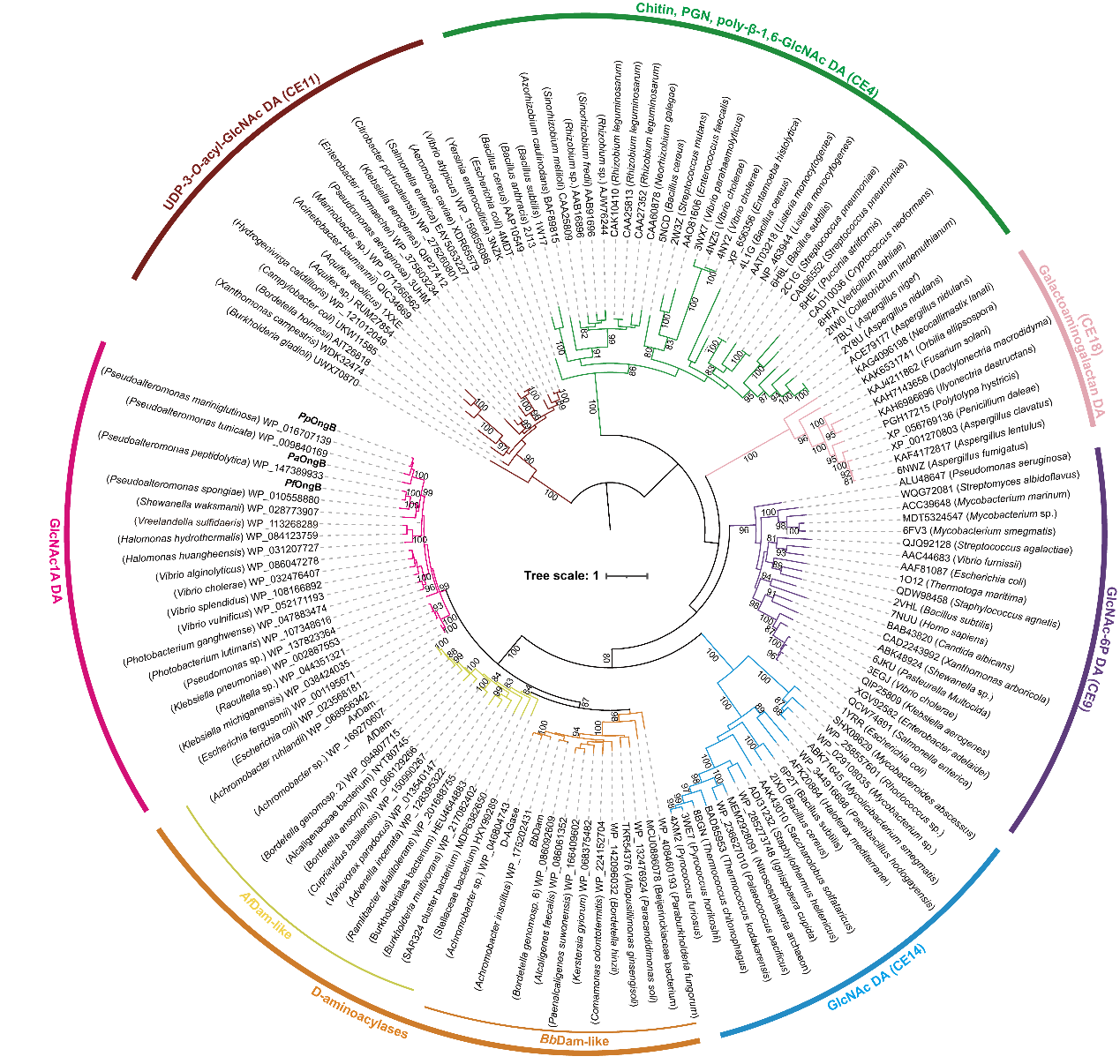


**Figure S8 A maximum likelihood phylogenetic tree of *Pp*OngB and other carbohydrate de-*N*-acetylases (a total of 150 protein sequences) constructed using** **IQ-TREE.** Bootstrap analysis of 1,000 replicates is conducted and values above 80 are shown. For each sequence, its source strain is indicated in parentheses.


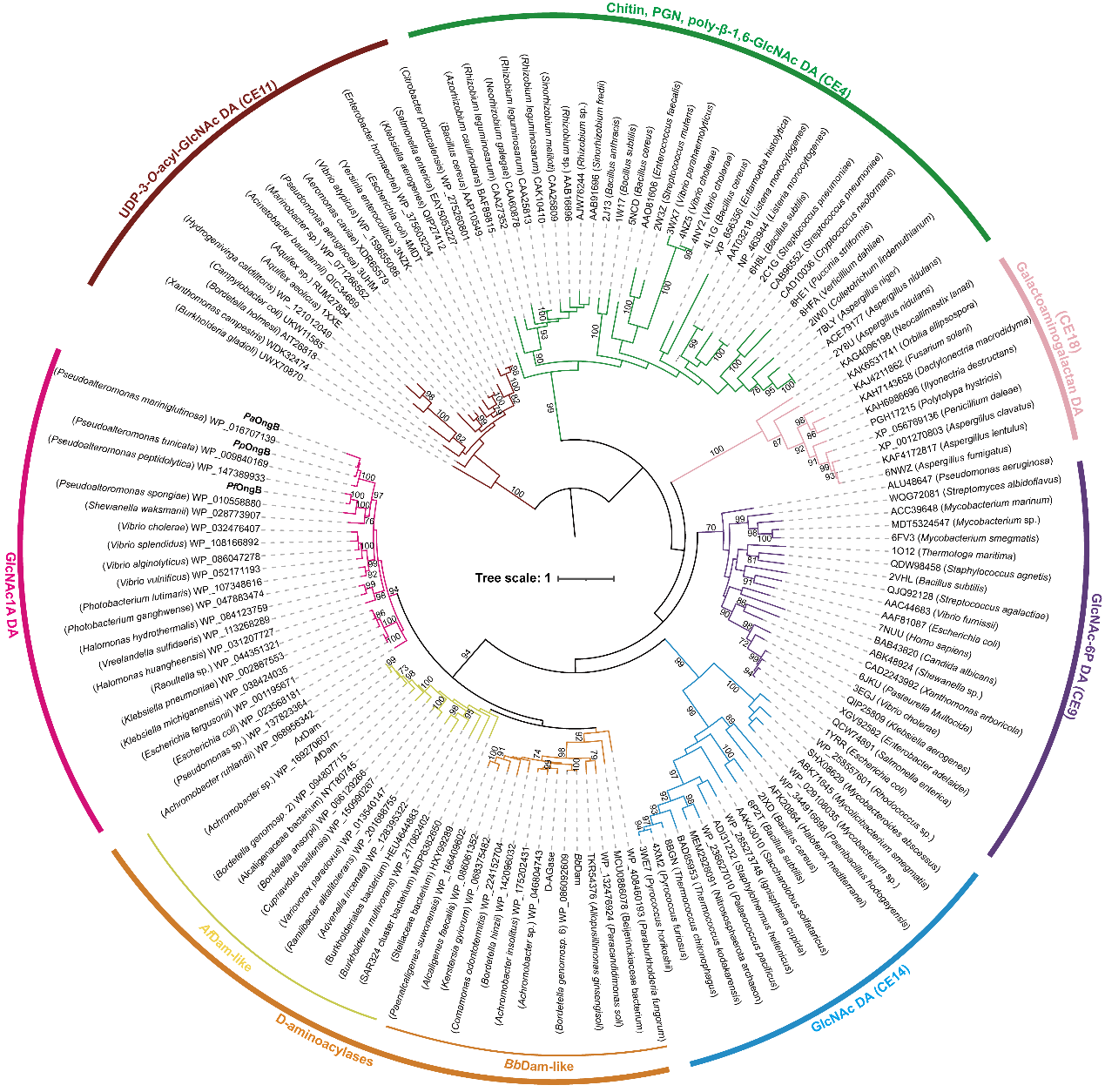


**Figure S9 A maximum likelihood phylogenetic tree of *Pp*OngB and other carbohydrate de-*N*-acetylases (a total of 150 protein sequences)** **constructed using RAxML.** Bootstrap values above 70 are shown. For each sequence, its source strain is indicated in parentheses.
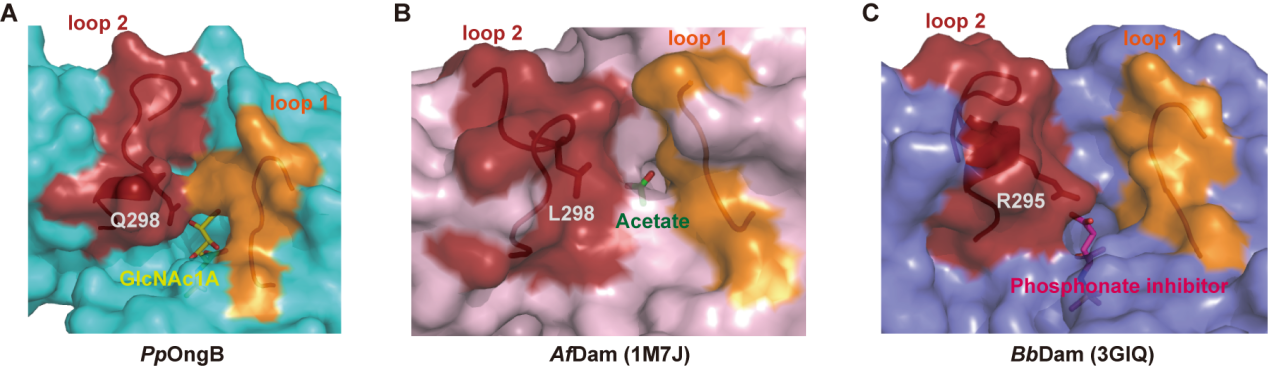


**Figure S10 Close-ups of the entrances of catalytic cavities of *Pp*OngB (A), *Af*Dam (B) and *Bb*Dam (C).** Loops 1 and 2 are colored in orange and firebrick, respectively. The bound substrate/product/inhibitor is shown as sticks in different colors.
